# Abuse‐Tolerant Electrolytes for Lithium‐Ion Batteries

**DOI:** 10.1002/advs.202003694

**Published:** 2021-03-18

**Authors:** Zhiqi Chen, Yunfeng Chao, Weihua Li, Gordon G. Wallace, Tim Bussell, Jie Ding, Caiyun Wang

**Affiliations:** ^1^ ARC Centre of Excellence for Electromaterials Science Intelligent Polymer Research Institute AIIM Facility Innovation Campus University of Wollongong Wollongong NSW 2500 Australia; ^2^ School of Mechanical, Materials, Mechatronic and Biomedical Engineering University of Wollongong Wollongong NSW 2522 Australia; ^3^ Defence Science and Technology Group Department of Defence Melbourne VIC 3207 Australia

**Keywords:** electrolytes, flame retardants, lithium‐ion batteries, redox shuttles, shear thickening

## Abstract

Safety issues currently limit the development of advanced lithium‐ion batteries (LIBs) and this is exacerbated when they are misused or abused. The addition of small amounts of fillers or additives into common liquid electrolytes can greatly improve resistance to abuse without impairing electrochemical performance. This review discusses the recent progress in such abuse‐tolerant electrolytes. It covers electrolytes with shear thickening properties for tolerating mechanical abuse, electrolytes with redox shuttle additives for suppressing electrochemical abuse, and electrolytes with flame‐retardant additives for resisting thermal abuse. It aims to provide insights into the functioning of such electrolytes and the understanding of electrolyte composition‐property relationship. Future perspectives, challenges, and opportunities towards practical applications are also presented.

## Graphical abstract

The addition of fillers/additives into liquid electrolytes has enabled the abuse‐resistant properties towards safe lithium‐ion batteries. This review presents three types of electrolytes that can provide the protection against the commonly encountered abuses, including shear thickening electrolytes to tolerate mechanical abuse, redox shuttle additive‐based electrolytes to suppress electrochemical abuse, and flame‐retardant additive based electrolytes to resist thermal abuse.

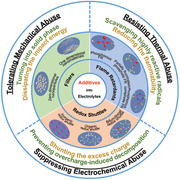

## 1 Introduction

To ease the concern with greenhouse gas emissions and natural resource depletion, sustainable and green energy sources (e.g., solar energy, wind energy, tidal energy) have been developed.^[^ ^1^ ^]^ The buffering of the intermittent nature of these renewable energy resources calls for the development of electrochemical energy storage systems.^[^ ^2^ ^]^ Currently lithium‐ion batteries (LIBs) are the most competitive energy storage system with high specific energy densities and stable/long term cycling performance.^[^ ^3^ ^]^ They have been dominantly and extensively applied in portable electronic devices, electric vehicles (BEVs), hybrid electric vehicles (HEVs), and plug‐in hybrid electric vehicles (PHEVs).^[^ ^4^ ^]^ In 2019, John B. Goodenough, M. Stanley Whittingham, and Akira Yoshino shared the Nobel Prize in Chemistry due to their significant contribution in the field of lithium‐ion batteries.^[^ ^5^ ^]^ LIBs are the most successful battery system developed to date and they currently dominate the battery market. The global LIB market was 23.5 billion in 2017 and estimated to be $71 billion by 2025, at an expected compound annual growth rate (CAGR) of 14.9% during the period from 2018 to 2025.^[^ ^6^ ^]^ However, improper operation of LIBs can cause a spontaneous violent reaction with an abrupt release of chemical energy leading to fires and explosions.^[^ ^4^, ^7^ ^]^ Fire or explosive incidents related to LIBs include a Tesla Model S,^[^ ^8^ ^]^ Samsung Galaxy Note 7,^[^ ^9^ ^]^ and the main battery of a Boeing airplane was damaged leading to an emergency landing during flight.^[^ ^10^ ^]^

**Figure 1:**
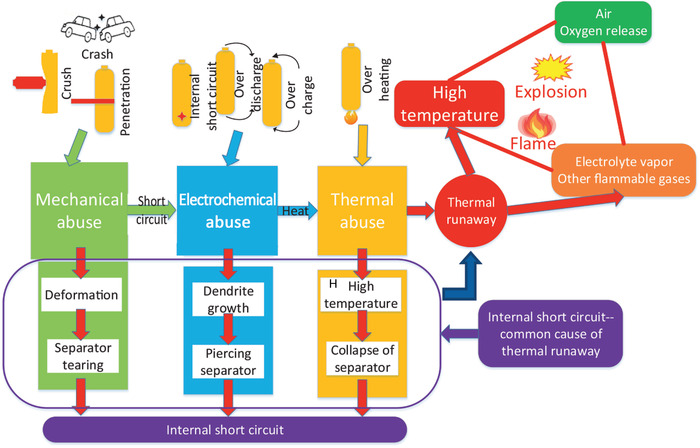
Schematic illustration of the LIB failure and the correlated abuse conditions. Reproduced with permission.^[^ ^11b^ ^]^ Copyright 2019, Elsevier Ltd.

The estimated failure rate of LIBs is less than 1 in 40 million under the recommended operating and storage conditions.^[^ ^11^ ^]^ However, this rate is dramatically increased under misuse/abuse conditions (e.g., mechanical abuse, electrochemical abuse, or thermal abuse), as illustrated in **Figure** **1**. Mechanical abuse includes the crushing, penetration or dropping causing cell deformation resulting in internal short circuit or/and electrolyte leakage to induce a fire and/or explosion.^[^ ^7^, ^8^, ^12^ ^]^ Electrochemical abuse from overcharging may induce the formation of dendritic lithium on the negative electrode^[^ ^7^, ^8^, ^11^, ^12^ ^]^ and the structural collapse and subsequent oxygen release on the positive electrode.^[^ ^8^, ^11^, ^12^, ^13^ ^]^ This causes a short circuit and the generated heat and gas from the electrolyte decomposition can trigger thermal runaway leading to fire and/or explosion. Thermal abuse involves use outside the thermal stability limits of LIBs and includes electrolyte decomposition and/or melting of separator which lead to thermal runaway following exothermic reactions.^[^ ^7^, ^8^, ^11^, ^12^, ^14^ ^]^

External protection measures have been used to mitigate against thermal runaway incidents. It includes the introduction of electronic safety devices within a battery system to decrease the possibility of flammable hazard and upgrade the safety such as safety vent,^[^ ^11^, ^12^, ^15^ ^]^ current interruption devices,^[^ ^11^, ^12^, ^16^ ^]^ temperature sensors,^[^ ^7^, ^11^, ^12^, ^17^ ^]^ and cooling packages.^[^ ^11^, ^12^, ^18^ ^]^ These methods inevitably introduce dead weight and volume with a subsequent lowering of the energy density.^[^ ^12a,d^ ^]^ In contrast, internal protection mechanisms rely on using intrinsically safe materials, and could provide “ultimate” solution for safe batteries.^[^ ^7^, ^12^ ^]^

There are four primary components in an LIB: cathode, electrolyte, separator, and anode. The electrolyte is regarded as the blood of a battery system transferring ions between positive and negative electrodes to complete the chemical energy‐electrical energy conversion.^[^ ^19^ ^]^ The dynamics of lithium ion transport dictate the operating temperature, rate capability, lifetime and the practical accessible capacity of LIBs.^[^ ^19^, ^20^ ^]^ There are three types of electrolytes, liquid electrolytes including ionic liquid, polymer electrolytes, and solid inorganic electrolytes.^[^ ^19^ ^]^ Polymer electrolytes, solid inorganic electrolytes and ionic liquid are generally considered as safe electrolytes.^[^ ^19^, ^21^ ^]^ There have been excellent reviews available on these electrolytes for safe LIBs: for polymer electrolytes,^[^ ^19^, ^22^ ^]^ solid inorganic electrolytes^[^ ^19^, ^22^, ^23^ ^]^ (e.g., sulfides,^[^ ^23l–o^ ^]^ oxides,^[^ ^23m–o^ ^]^ halides,^[^ ^23o^ ^]^ and closo‐borates^[^ ^23o^ ^]^), and ionic liquid electrolytes.^[^ ^19^, ^22^, ^24^ ^]^ Generally speaking, solid polymer electrolytes commonly suffer from low ionic conductivity, a balance needs to be struck between mechanical strength and ionic conductivity for gel polymer electrolytes.^[^ ^19^, ^25^ ^]^ The low ionic conductivity, poor interface with electrodes and difficulty in large scale fabrication are currently limiting the practical use of solid inorganic electrolytes in LIBs.^[^ ^19^, ^26^ ^]^ The practical application of ionic liquid electrolytes is hindered by prohibitive cost and inferior performance with low‐rate capacity and poor cycle performance at room temperature.^[^ ^19^, ^24^ ^]^

Commonly used liquid electrolytes in LIBs industry consist of flammable and volatile organic carbonates and lithium salts. They can offer high ionic conductivity and good compatibly with electrodes forming a stable solid‐electrolyte interphase (SEI), and deliver excellent battery performance.^[^ ^27^ ^]^ The introduction of small amount of additives into liquid electrolytes is a facile and cost‐effective way to improve safety without impairing the original properties.^[^ ^22^, ^24^, ^28^ ^]^ Zhang^[^ ^28^ ^]^ in 2006 and Haregewoin et al.^[^ ^29^ ^]^ in 2016 presented excellent comprehensive reviews on electrolyte additives for LIBs with reference to battery performance improvement and battery safety enhancement under the overcharge and thermal abuse conditions during the last two decades. Kalhoff et al.^[^ ^22c^ ^]^ in 2015 reviewed the additives used in electrolytes for safer LIBs against the overcharge and thermal abuse. Shu et al.^[^ ^30^ ^]^ recently presented the development in shear thickening electrolytes (STEs) to better tolerate mechanical abuse. Here we present a comprehensive review summarizing the additives/fillers used in the electrolytes that can provide the protection against all three common types of abuse: mechanical abuse, electrochemical abuse, and thermal abuse. They are STEs, electrolytes with redox shuttle additives, and electrolytes with flame‐retardant additives. We define them as abuse‐resistant electrolytes. We will focus on discussing the working principle of these electrolytes, the fillers/additives used to facilitate the realization of abuse‐resistant properties, and the correlation between the abuse‐resistant properties and electrochemical performance.

## 2 Abuse‐Tolerant Electrolytes

### 2.1 Shear Thickening Electrolytes

#### 2.1.1 Shear Thickening Effects

Shear thickening is one type of non‐Newtonian behavior where the stress (i.e., viscosity in the research field of rheology) increases non‐linearly with the increase in shear rate, and the increase is rather dramatic and transient.^[^ ^30^, ^31^ ^]^ Shear thickening fluids (STFs) consist of rigid and colloidal particles in an inert medium solvent, in which the shear viscosity can drastically increase beyond a critical shear rate.^[^ ^30^, ^32^ ^]^ This behavior can be used to provide protection against dynamic impacts, and thus STFs have been applied in liquid body amour,^[^ ^33^ ^]^ as damper systems for shock absorbing,^[^ ^34^ ^]^ and as a tamp in controlled pulse fracturing (CPF).^[^ ^35^ ^]^

**Figure 2:**
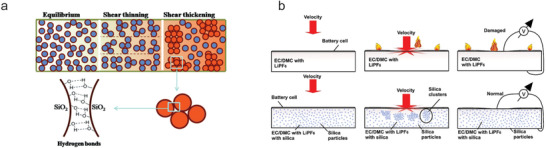
a) Schematic illustration of the changes in microstructure of STFs for the transition from equilibrium to shear thickening and the hydrogen bonding interaction at surface between particles in a hydrocluster. b) Schematic demonstration of the protective mechanism of STEs under impact test. a) Reproduced with permission.^[^ ^37^ ^b]^ Copyright 2016, The Royal Society of Chemistry. b) Reproduced with permission.^[^ ^41^ ^]^ Copyright 2013, Springer Nature.

Different mechanisms (i.e., hydrocluster, order disorder transition, and dilatancy) have been developed to describe the operation of STFs.^[^ ^32b^ ^]^ The hydrocluster mechanism has been experimentally confirmed,^[^ ^36^ ^]^ where an energy from the shear stress suddenly surpasses the repulsive forces between neighboring particles and forces them to come together as larger hydroclusters.^[^ ^32^ ^]^ Taking silica fillers as an example (**Figure** **2**a), repulsive forces (e.g., electrostatic forces, steric hindrance, and solvent forces) result in particle–particle repulsion during the Newtonian/equilibrium and shear thinning regime.^[^ ^32^, ^37^ ^]^ Beyond the critical shear rate, the interparticle repulsive forces become lower than the hydrodynamic lubrication force resulting in the formation of large aggregates through strong hydrogen bonding between hydroxyl groups on the silica surface.^[^ ^30^, ^32^ ^]^ These hydroclusters are broken up and return to the scattered state as a stable suspension after the removal of shear stress.

The impact resistant capability of STFs can be explained in that the induced shear thickening behavior buffers mechanical impact. Figure 2b shows the protective mechanism of STFs under dynamic impact (i.e., a low energy impact in a LIB). The battery system with common commercial electrolytes, for example, LiPF_6_ in EC/DMC, suffers a large deformation under the impact and the separator fails to isolate the electrodes; as a result, the temperature in the system rapidly increases which may ignite the electrolytes and the battery is damaged. Conversely, the electrolyte with shearing thickening behavior turns into a solid‐like state and dissipate the impact energy due to the formation of silica particle clusters when using silica as additives, and thus effectively improve the rigidity of the battery system and maintain the battery functioning. That is to say, shear thickening behavior can provide the protection against dynamic impact due to the buffering feature. The perfect hunting ground for this concept is from the “soggy sand” electrolyte because of their similarity, where both electrolytes are composed of particles and carrier fluids.^[^ ^38^ ^]^

Discontinuous shear thickening (DST) refers to a phenomenon in STFs that a flow induced bunch aggregation causes the jamming and behaves like a solid.^[^ ^39^ ^]^ Generally, DST is coincident with a disruption of the viscosity at the critical shear rate, and related to the volume fraction of fillers.^[^ ^39a^ ^]^ The particle volume fraction is defined as the fraction of total STFs volume by filler volume, and is the major factor affecting the rheology of STFs. The critical volume fraction required to realize the shear thickening behavior increases with the decreased aspect ratio (AR) of filler particles.^[^ ^40^ ^]^ With the increasing of volume fraction, the shear thickening behavior occurs at a lower shear rate region and the viscosity increases dramatically.

#### 2.1.2 Shear Thickening Electrolyte

The addition of fillers (e.g., fumed silica nanoparticles) into liquid electrolytes forms a new type of electrolyte with shear thickening behavior: shear thickening electrolytes (STEs). A simple agitating process was involved in the adding of fillers into liquid electrolytes and a sonication process was used to improve homogeneity of the dispersion in some cases. STEs can also be regarded as the adding of lithium salts into STFs. This type of electrolyte possesses the shear thickening and ionically conductive properties. Just as STFs, STEs in LIBs can also provide the protection against mechanical abuse by utilizing the shearing thickening effect.

**Figure 3:**
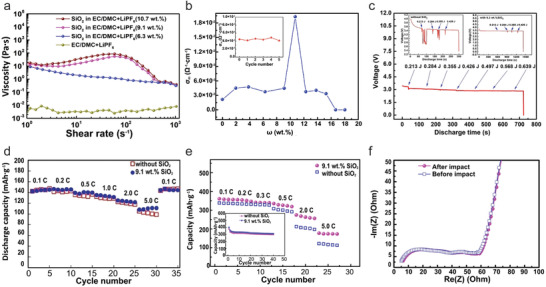
Rheological and electrochemical properties of the electrolytes containing different amount of silica fillers. a) Rheological profile of the electrolytes (1 m LiPF_6_ in EC/DMC) containing different amount of SiO_2_: 0, 6.3, 9.1, and 10.7 wt%. b) Ionic conductivity as function of weight fraction (*ω*) of fumed silica for STEs. c) Discharge curves of LiFePO_4_ electrodes in half‐cells with electrolytes containing SiO_2_ of 0, 9.1, and 6.3 wt% under different energy impacts during the discharge. d) Rate performance comparison of LiFePO_4_ electrodes in half cells with STE and standard electrolytes. e) Rate performance comparison of graphite electrodes in half cell with STE and standard electrolytes; Inset, cycling performance. f) Nyquist plots of graphite electrode in STEs before and after the impact. Reproduced with permission.^[^ ^41^ ^]^ Copyright 2013, Springer Nature.

The concept of STE was first developed by Ding et al.,^[^ ^41^ ^]^ where they demonstrated the dispersing of fumed silica nanoparticles into conventional electrolyte (1 m LiPF_6_ in EC/DMC)and produced a shear thickening effect with outstanding resistance to kinetic impact. The STE with 9.1 wt% silica exhibited a shear thickening behavior, and a better ionic conductivity than the standard commercial electrolyte (**Figure** **3**a,b). The coin cell composed of LiFePO_4_/STEs/Li sustained a dynamic impact of 0.568 J with a stable discharge voltage profile, while the cell with the electrolytes with 0 and 6.3 wt% silica short circuited under a low impact energy of 0.426 J (Figure 3c). The full cell of graphite anode and LiCoO_2_ cathode showed similar effects to the applied impact energy. The LiFePO_4_ half cells and graphite half cells with STEs demonstrated slightly better rate performance than the cells with commercial electrolytes (Figure 3d,e). No obvious difference was presented on the Nyquist plots of the half‐cell with graphite anode and STEs before and after the impact (Figure 3f), which verified the reversibility of STEs. Namely, STEs can reversibly switch between liquid and semisolid or solid phase. This is a pioneer work, and first demonstrated that the use of STEs can improve the impact resistance without compromising the battery performance.

#### 2.1.3 Fillers Used in STEs

##### Zero‐Dimension (0D) Nanoparticles

**Figure 4:**
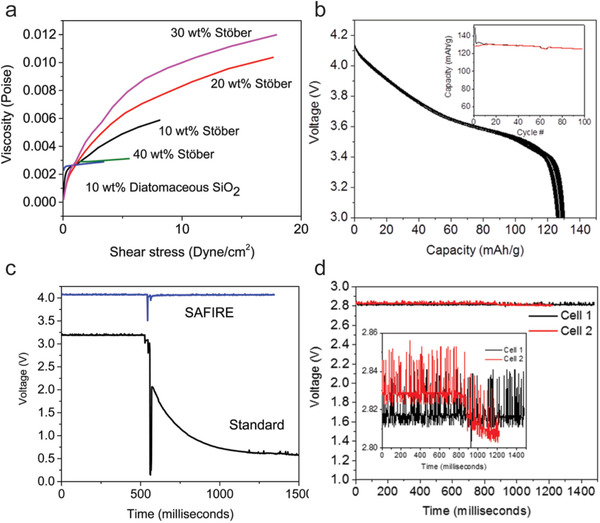
a) Rheological diagram of viscosity as a function of shear stress for different shear thickening fluids. b) Discharge curves of a cell with the SAFIRE electrolyte; Inset, cycling performance. c) Voltage profiles of cells with PEEK scaffold separator and SAFIRE or standard electrolyte upon the impact. d) Discharge voltage stability of two pouch cells with the SAFIRE electrolyte under the impact energy of 5.65 J. Reproduced with permission.^[^ ^42a^ ^]^ Copyright 2017, American Chemical Society.

To date, 0D silica nanoparticles are still the most used additives in lithium‐ion battery electrolytes to deliver the shear thickening effect when subjected to the shear stress/impact energy.^[^ ^41^, ^42^ ^]^ Veith et al.^[^ ^42a^ ^]^ investigated the rheological properties of various silica particles based on polydispersity index. The STE with a maximum shear thickening response was achieved by dispersing 30 wt% Stöber silica with low polydispersity (<0.01) in the electrolyte (1.2 m LiPF_6_ in 3:7 wt% EC/DMC) as shown in **Figure** **4**a. They named such electrolytes as safe impact resistance electrolytes (SAFIRE), as colloidal silica particles could form particle aggregations as a solid barrier in the electrolyte upon the impact. The full cell of nickel manganese cobalt oxide (NMC)/SAFIRE/graphite demonstrated stable and good cycling performance over 100 cycles (Figure 4b). The cell with a polyether ether ketone (PEEK) mesh separator and SAFIRE experienced a sudden voltage drop upon the impact and then returned to the original value, while the cell with standard electrolytes immediately short‐circuited and the voltage dropped a lot (Figure 4c). The pouch cell with SAFIRE endured a 5.65 J energy impact from a steel ball and displayed a slightly fluctuational change in voltage (Figure 4d), in contrast to the obvious voltage drop with standard electrolyte. They also developed a sterically stabilized silica with the modification of poly (methyl methacrylate) (PMMA) brushes as fillers for STEs, which showed good shear thickening effect with a decreased possibility of forming silica sediment.^[^ ^42b^ ^]^ This kind of STE showed stable viscosity and conductivity over 24 h in contrast to the decayed viscosity and conductivity for the STE with unmodified silica. The covalent bounds between PMMA brushes and silica nanoparticles surface groups endows the STE with long‐term stability. The NMC/PMMA‐silica based STE/graphite full cell also demonstrated a better electrochemical performance compared to the cells with untreated silica based STEs and commercial electrolyte.

##### One Dimensional (1D) Nanomaterials

**Figure 5:**
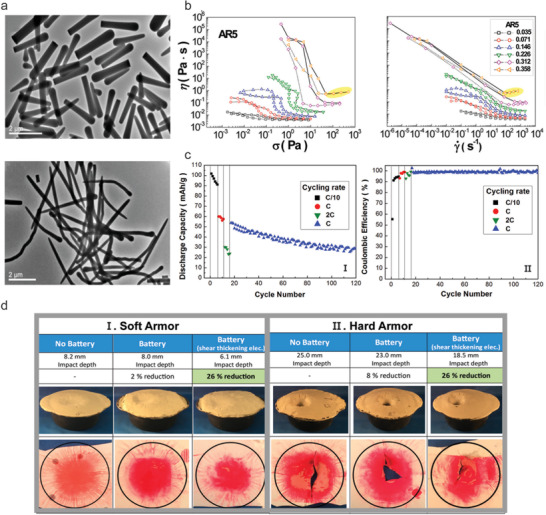
a) TEM images of AR5 (top) and AR24 silica nanorods (bottom). b) Rheological results of AR5 silica nanorods in the standard electrolyte: I) viscosity (*ƞ*) versus shear stress (*σ*); II) viscosity (*ƞ*) versus shear rate ($\dot{\gamma }$). Yellow shaded area is the shear thickening region. c) Cycling profile of discharge capacity (I) and coulombic efficiency (II) for the NMC/graphite CR2032 full cell with the AR5 nanorods (*Φ* = 0.33) added EC/EMC/LiTFSI electrolyte at different current rates. d) Results of ballistic impact test for I) soft armor and II) hard armor under the conditions of without battery, with battery containing EC/EMC/LiTFSI electrolyte, and with battery containing AR5 nanorods (*Φ* = 0.358) in EC/EMC/LiTFSI electrolyte. Reproduced with permission.^[^ ^43^ ^]^ Copyright 2018, American Chemical Society.

The volume fraction of fillers required for inducing the shear thickening decreases with the increase in AR, which is attributed to the lower packing density of particles.^[^ ^43^ ^]^ Particles with lower volume fraction need lower hydrodynamic forces to overcome the repulsive forces due to the larger distance between neighboring particles and no restriction of particle motions in the suspensions.^[^ ^40^ ^]^ Particle dimensions and interparticle forces are the two key parameters regulating shear thickening behavior.^[^ ^40^, ^43^ ^]^ With the increase of AR, the particle anisotropy increases resulting in the poor particle packing in the unaligned state leading to the generation of shear thickening effect at a lower shear loading.^[^ ^43^ ^]^ Hence, silica nanorods or fiber type fillers with high ARs show a shearing thickening behavior at lower critical volume fractions compared to silica nanoparticles.^[^ ^44^ ^]^ Anisotropic silica nanorods with an AR of 24 had a much lower volume fraction of 0.146 than the 0.358 for nanorods with an AR of 5.^[^ ^43^ ^]^ Silica nanorods with a controllable AR from 2 to 24 were obtained by growing silica tetraethyl orthosilicate (TEOS) on different templates (e.g., droplets formed with sodium citrate, poly(vinylpyrrolidone), ammonium hydroxide, ethanol, or pentanol) in the emulsion. These nanorods had a relatively constant diameter with controlled length (**Figure** **5**a). Shear thickening behavior appeared for AR5 silica nanorods, marked as yellow shade area in Figure 5b. Moreover, the STE with 1D fillers of large ARs may present a high ionic conductivity because of the more void available for ion transportation.^[^ ^45^ ^]^ The cell composed of NMC cathode, STEs containing silica nanorods with an AR of 5, and graphite anode demonstrated a nominal capacity of 148 mAh g^−1^, a better capacity retention rate compared to the cell in STE with untreated fumed silica (Figure 5c), and a better protection against the ballistic test with a 26% reduction in impact depth (Figure 5d). The impact depth was the shallowest in the case of batteries with STEs behind both soft and hard amour. In addition, there was a noticeable difference of less pink color for evidencing the impact force distribution in the pressure‐sensitive films in which had a battery with STE.

**Figure 6:**
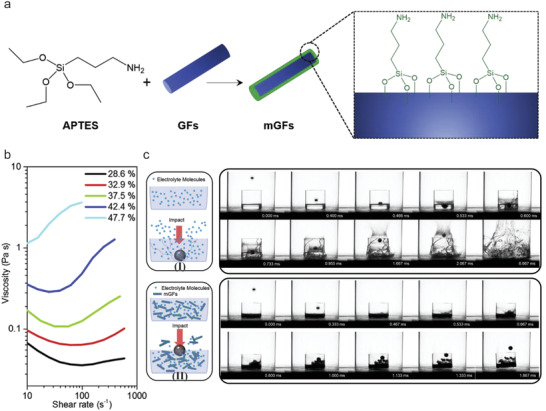
a) Schematic fabrication of APTES modified glass fibers (GFs). b) Rheological results of viscosity versus shear rate for STEs with different filler volume fractions. c) Results of the high speed (79 m s−^1^) impact test onto the conventional electrolytes (I) and STE (II) in a glass. Reproduced with permission.^[^ ^46^ ^]^ Copyright 2019, Elsevier B.V.

Another low‐cost 1D material, (3‐aminopropyl) triethoxysilane (APTES) modified glass fiber fillers with amine ending group, have been utilized in STEs (**Figure** **6**a). Fillers with a high AR of 5 to 10 enabled shear thickening behavior (Figure 6b) at low volume fractions (28.6%).^[^ ^46^ ^]^ This STE provided good protection against mechanical abuse, as verified from the stainless‐steel ball dropping test onto the regular electrolyte and STEs in a glass container (Figure 6c). The ball bounced back when dropped onto the STE in the glass container as recorded by a high‐speed camera (Figure 6c ‐II). In sharp contrast for regular electrolytes, the glass container was broken on impact (Figure 6c‐I). Electrochemical cells containing this STE demonstrated excellent cyclability, a capacity retention of 95.2% over 500 cycles for a LiFePO_4_ half‐cell and a 94.4% capacity retention after 100 cycles for the lithium iron phosphate (LFP) and lithium titanate (LTO) full cell. Moreover, the LFP‐LTO pouch cells with STE tolerated an impact energy of 2.04 J, as reflected by a stable discharge voltage in contrast to a larger voltage drop of 6.2% for the cell with standard electrolyte.

An overview of shearing thickening electrolytes is summarized in **Table** **1**. It includes fillers used in and the composition of STEs, the impact‐resistant properties, and electrochemical performance in LIBs.

**Table 1:** Overview of shear thickening electrolytes for lithium‐ion batteries

Fillers		Composition of STEs	Cell properties		Ref.
Type	Materials		Impact‐resistant property	Electrochemical performance	
0D nanoparticles	Fumed silica	6.3 wt% SiO_2_ in 1 m LiPF_6_ (solvent, 1:1 EC^a^ ^)^/DMC^b^ ^)^)	The fabricated coin cell sustained a dynamic impact tolerance of 0.568 J.	Compared to the cells with conventional electrolytes, better rate capacity displayed for the LiFePO_4_/Li cell with STEs and a slightly better rate performance and a higher reversible capacity for the graphite/Li cell.	^[^ ^41^ ^]^
	Stöber silica	30 wt% stöber silica in 1.2 m LiPF_6_ (solvent, 3:7 wt% EC/DMC; SAFIRE)	The fabricated pouch cell endured a 5.65 J energy impact.	The NMC^c^ ^)^/SAFIRE/graphite full cell demonstrated stable discharge and charge performance over 100 cycles.	^[^ ^42a^ ^]^
	PMMA^d^ ^)^‐silica	30 wt% PMMA‐silica in 1.2 m LiPF_6_ (solvent, 3:7 wt% EC/DMC)	N/A	The NMC/PMMA‐silica based STE/graphite cell demonstrated better electrochemical performance than the NMC/bare silica based STEs/graphite cell.	^[^ ^42b^ ^]^
1D nanomaterials	Silica nanorods	33 vol% AR5^e^ ^)^ silica nanorods in 1 m LiTFSI^f^ ^)^ (solvent, 1:1 EC/EMC^g^ ^)^)	The pouch cell showed better protection during the ballistic test with a 26% reduction in impact depth.	The NMC/STE/graphite showed a nominal capacity of 148 mAh g^−1^ and a better capacity retention rate.	^[^ ^43^ ^]^
	APTES^h^ ^)^ modified glass fiber (mGFs)	37.5 vol% mGFs in 1 m LiPF_6_ (solvent, EC/DMC)	The fabricated pouch cell tolerated an impact energy of 2.04 J.	With STE as the electrolyte, the LiFePO_4_/Li cell showed a capacity retention of 95.2% over 500 cycles, and LiFePO_4_/LTO^i^ ^)^ cell demonstrated a 94.4% capacity retention rate after 100 cycles.	^[^ ^46^ ^]^

^a)^EC, ethylene carbonate

^b)^DMC, dimethyl carbonate

^c)^NMC, nickel manganese cobalt oxide

^d)^PMMA, poly(methyl methacrylate)

^e)^AR5, aspect ratio of 5

^f)^LiTFSI, lithium bis(trifluoromethanesulfonyl)imide

^g)^EMC, ethyl methyl carbonate

^h)^APTES, (3‐aminopropyl) triethoxysilane

^i)^LTO, lithium titanate.

### 2.2 Electrolytes with Redox Shuttle Additives

#### 2.2.1 Redox Shuttle Effects

Overcharging is one of the most common electrochemical abuse scenarios threatening safe operation of LIBs. Overcharging may trigger chemical and electrochemical decomposition reactions of electrodes and electrolyte, generating gaseous products (e.g., O_2_, CO_2_, CO) accompanied with a rapid temperature upsurge that may lead to fire and explosion.^[^ ^7^, ^22^, ^47^ ^]^ Overcharging conditions result in excessive lithium de‐intercalation, resulting in the crystal structure collapse of cathode material at the positive electrode, the dissolution of the copper current collector together with significant formation of dendritic lithium at the negative electrode, which eventually causes short‐circuiting.^[^ ^7^, ^48^ ^]^ In addition, a thick and uneven cathode–electrolyte interface (CEI) film is formed when extending the upper cut‐off voltage limit because of the excessive decomposition of electrolyte. It is also accompanied by the dissolution of transition metals, with subsequent electrolyte decomposition resulting in a thick CEI layer, degrading the charge–discharge efficiency and battery life.^[^ ^49^ ^]^ To mitigate the overcharge‐induced decomposition reactions, a small amount of redox shuttle molecules can be added into the electrolyte. This provides overcharge protection by avoiding reactions between cell components, gas release, and the rapid temperature increase of the cell at high voltages,^[^ ^47^, ^50^ ^]^ and is considered as an economic and efficient approach.

**Figure 7:**
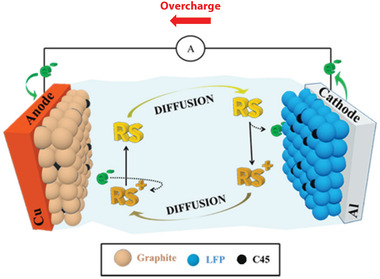
Schematic illustration of the redox shuttle effect during the overcharging in a battery system with graphite anode and carbon‐coated lithium iron phosphate cathode. The oxidized materials are diffused and reduced at the anode surface, and the reduced materials are oxidized to consume the overcharge current. Consequently, the charge potential can be maintained at the potential of redox shuttle molecules. Reproduced with permission.^[^ ^50s^ ^]^ Copyright 2018, American Chemical Society.

In general, redox shuttle additives can be reversibly oxidized/reduced at a potential slightly higher than the end‐of‐charge potential of positive electrodes. This can shunt the overcharge current and restrain the cathode potential at the redox potentials of the shuttle molecules. As illustrated in **Figure** **7**, during the overcharging of LIBs, the redox shuttle molecule (RS) is oxidized to its radical cation (RS^+^) at the cathode/electrolyte interface, mitigating the oxidation and decomposition of electrolyte (Equation 1). The oxidized shuttle (RS^+^) diffuses across the electrolyte to the anode and is reduced back (Equation 2). The oxidation–diffusion–reduction cycle occurs continuously during overcharging owing to the highly reversible nature of the redox shuttles; thus, the end‐of‐charge potential of the cathode is locked at the intrinsic oxidation potential of the redox shuttle molecules.^[^ ^47^ ^]^

(1) $$\mathrm{Cathode}:\;\mathrm{RS}\to RS^{+}+ne^{-}$$

(2) $$\mathrm{Anode}:\;RS^{+}+ne^{-}\to \mathrm{RS}$$

The ideal redox shuttle should fulfill the following characteristics in order to efficiently prevent the overcharging:

1) The oxidation potential is slightly higher than the end‐of‐charge potential of positive electrodes, but lower than the oxidative decomposition potential of the electrolyte;^[^ ^7^, ^22^, ^51^ ^]^ otherwise, it may cause the incomplete charging of the battery and decreases the efficiency or oxidizes the electrolyte resulting in safety issues.
2) The molecules should be highly electrochemically reversible.^[^ ^7^, ^22^, ^51^ ^]^
3) The molecules should have good solubility with high mobility and high diffusion coefficient in the electrolyte to deliver the overcharge protection at high current density.^[^ ^7^, ^22^, ^50^, ^51^ ^]^
4) The molecules should be chemically, electrochemically, and thermally stable and inert towards all the components of a LIB at all stages. This is of vital importance in providing long term overcharge protection.^[^ ^7^, ^22^, ^51^, ^52^ ^]^

During the overcharge tests, LIBs with different types of cathode materials with such type of electrolytes can sustain the charge–discharge cycles, where the cell voltage is maintained beyond a safe voltage (i.e., ≈4.0 V for olivine type materials (LiFePO_4_), ≈4.5 V for layered oxide type materials (LiCoO_2_), ≈4.5 V for spinel oxide type materials (LiMn_2_O_4_)) under 100% overcharge (charged to 200% of nominal charge capacity) per cycle.^[^ ^47^, ^53^ ^]^ The limiting shuttle current (*I*_max_) in terms of overcharge protection at high voltage and high current density depends on the electrode area, diffusion coefficient, and concentration of redox shuttle molecules in the electrolyte, and the distance of interelectrode according to Equation (3).^[^ ^54^ ^]^

(3) $$I_{\max}=nFADC/L\;$$

where *n* stands for the number of charges carried by the redox shuttle molecule (normally *n* is equal to 1), *F* is Faraday's constant, electrode area (*A*) and the electrode spacing (*L*) is a fixed geometry in the battery system, and the diffusion coefficient (*D*) is specified by the physical and chemical properties of the redox shuttle and solvent. Hence, the concentration of the redox shuttle (*C*) is the only variation and plays an important role in determining the limiting shuttle current *I*_max_ of the redox shuttle system.

#### 2.2.2 Inorganic Redox Shuttle Additives

Behl et al.^[^ ^50a,b^ ^]^ first reported the use of the redox couple I^−^/I_3_^−^ (iodide to tri‐iodide) with an oxidation potential at ≈3.25 V (versus Li^+^/Li) as a redox shuttle in LiAsF_6_/tetrahydrofuran (THF) electrolyte. In an electrochemical quasi‐reversible system, the I^−^/I_3_^−^ shuttle demonstrated a lower oxidation potential than THF, and proved useful for the overcharge protection of LIB with a LiAsF_6_/THF electrolyte. Thereafter, Br^−^/Br_3_^−^ (bromide to tri‐bromide) shuttle at ≈3.78 V (versus Li^+^/Li) was investigated for the overcharge protection of the 3 V LIBs.^[^ ^50c^ ^]^ However, bromide ion underwent a stepwise oxidation in LiAsF_6_/THF electrolyte to tribromide ion and bromine which induced the polymerization of THF. Moreover, these two kinds of halide‐based redox shuttles are not practically applicable in LIBs owing to their high reactivity and high volatility in oxidized halogen forms.^[^ ^7^, ^22^ ^]^

#### 2.2.3 Organic and Organometallic Redox Shuttle Additives

The metallocene family (i.e., ferrocene and organometallic ferrocene derivatives) have been investigated as redox shuttle additives.^[^ ^50d,e^ ^]^ Ferrocene is electrochemically reversible and can form stable ferrocenium cation via one‐electron oxidation.^[^ ^47^ ^]^ Golovin et al.^[^ ^50e^ ^]^ investigated eleven ferrocene derivatives with different substituents such as carbamylferrocene, carbomethoxyferrocene, methoxymethyl ferrocene, and (dimethylaminomethyl)ferrocene. These molecules provide a redox potential in the range of 3.2–3.5 V (versus Li/Li^+^) for overcharge protection without any negative effect on the cycle life of Li/Li*_x_*MnO_2_ cells, in which the shuttle voltage was close to the cathode potential. The derivative *N,N*‐DMAMF (dimethylaminomethylferrocene) was identified as the best candidate regarding to the high shuttle potential of 3.435 V and long cycle life.

Dahn et al.^[^ ^50f–h^ ^]^ reported that an aromatic compound with two methoxy groups and methoxy‐substituted benzenes, 2,5‐di‐*tert*‐butyl‐1,4‐dimethoxybenzene (DDB), provided a reversible and stable redox reaction at ≈4 V when these methoxy groups are at the ortho and para positions. DDB has rigid molecular structure with excellent electrochemical reversibility and stability. It provided a reversible redox potential of 3.9 V (versus Li/Li^+^) and more than 200 cycles of 100% overcharge per cycle at C/2 and 1C rate for LiFePO_4_ cathode in LIBs. However, it was of low solubility in carbonate‐based electrolyte due to its low polarity induced from symmetric structure. Isomers of DDB, 3,5‐di‐*tert*‐butyl‐1,2‐dimethoxybenzene (DBDB),^[^ ^50i^ ^]^ 4,6‐di‐*tert*‐butyl‐1,3‐benzodioxole (DBBD1) and 5,7‐di‐*tert*‐butyl‐1,4‐benzodioxin (DBBD2),^[^ ^50j^ ^]^ and 4‐*tert*‐butyl‐1,2‐dimethoxybenzene (TDB),^[^ ^50k^ ^]^ had high solubility in organic electrolytes and improved redox potential of 4.1–4.2 V (versus Li/Li^+^) but with much lower cycling performance, no more than 50 cycles of 100% overcharge per cycle. DDB could also be modified via introducing more‐electron‐withdrawing trifluoroethoxy groups or diethylphosphate groups to the methoxy moiety, such as 1,4‐di‐*tert*‐butyl‐2,5‐bis(2,2,2‐trifluoroethoxybenzene) (DBTFB, redox potential of 4.25 versus Li/Li^+^)^[^ ^50l^ ^]^ and tetraethyl‐2,5‐di‐*tert*‐butyl‐1,4‐phenylene diphosphate (TEDBPDP, redox potential of 4.8 versus Li/Li^+^).^[^ ^50m^ ^]^ These compounds were susceptible to anode reduction due to the formation of radical cations from the electron withdrawing group. TEDBPDP provided a redox shuttle potential at 4.8 V versus Li/Li^+^ and it is suitable for high potential cathode materials (e.g., LiCoO_2_, LiMn_2_O_4,_ Li_1.2_Ni_0.15_Co_0.1_Mn_0.55_O_2_) due to the introduction of diethylphosphate groups which strongly withdraw electron to increase the potential. DDB can also be modified via substituting the di‐*tert*‐butyl groups but retaining the methoxy group. The formed 1,4‐bis[bis(1‐methylethyl)phosphinyl]‐2,5‐dimethoxy‐benzene (BPDB) displayed a high redox potential of 4.5 versus Li/Li^+^.^[^ ^50n^ ^]^ However, BPDB was vulnerable to decomposition on the anode due to the increased electron affinity of the molecule leading to an inferior battery performance. All the above redox shuttle additives (modified DDB) have the delocalization of positive charges in their aromatic ring after the oxidative reaction, resulting in the steric protection for minimizing the formation of reactive sites.^[^ ^29^ ^]^ However, they are not comparable to DDB in terms of electrochemical stability due to the functional groups which cause the subsequent side reaction of the redox shuttle radical cation for easier decomposition.

**Figure 8:**
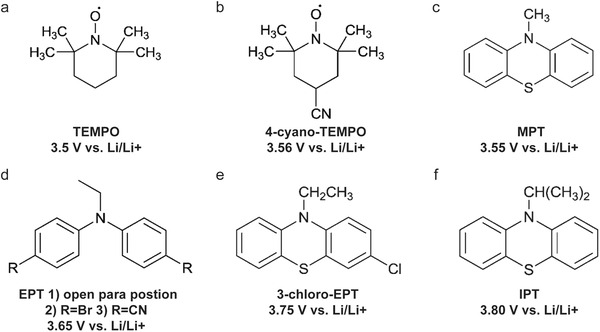
Chemical structure of redox shuttle additives: a) TEMPO; b) 4‐cyano‐TEMPO; c) MPT; d) EPT; e) 3‐chloro‐EPT; and f) IPT. Reproduced with permission.^[^ ^29^ ^]^ Copyright 2016, Royal Society of Chemistry.

Dahn and co‐workers discovered another type of stable redox shuttle additive systems, 2,2,6,6‐tetramethylpiperinyloxide (TEMPO, **Figure** **8**a) and derivatives of TEMPO with a five‐membered ring;^[^ ^50o^ ^]^ and phenothiazine (PT) derivatives.^[^ ^50p^ ^]^ Both these two systems displayed a redox potential in the range from 3.5 to 3.7 V (versus Li/Li^+^). TEMPO family was relatively stable shuttles and showed long cycling performance (more than 100 cycles) at 100% overcharge. For instance, TEMPO with a redox potential of 3.5 V versus Li/Li^+^ sustained 124 cycles of 100% overcharge per cycle, 4‐methoxy‐TEMPO could survive from 133 cycles of 100% overcharge per cycle, and 4‐cyano‐TEMPO (Figure 8b) provided 158 cycles of 100% overcharge per cycle. However, TEMPO and the derivatives have relatively low redox potentials and TEMPO^+^ cation was not very stable.^[^ ^55^ ^]^ Moreover, the TEMPO redox system cannot act as a stable redox shuttle additive in Li/Li_4_Ti_5_O_12_ cells, as the insertion‐extraction of lithium ions can be hindered by the passive layer formed on the Li_4_Ti_5_O_12_ electrode due to the self‐oxidation and reduction of TEMPO.

An alternative group of redox additives with comparable current shunting effects to DDB are phenothiazine (PT) derivatives, such as 10‐methylphenothiazine (MPT, Figure 8c) with a redox potential of 3.55 V versus Li/Li^+^; 10‐ethylphenothiazine (EPT, Figure 8d) having a redox potential of 3.65 V versus Li/Li^+^; 3‐chloro‐10‐methylphenothiazine (3‐chloro‐EPT, Figure 8e) with a redox potential of 3.75 V; and 10‐isopropylphenothiazine (IPT, Figure 8f) showing a redox potential of 3.80 V versus Li/Li^+^.^[^ ^50p,t^ ^]^ The LiFePO_4_/Li_4/3_Ti_5/3_O_4_ cells with MPT in 0.5 m lithium bis(oxalato)borate (LiBOB) based electrolyte and 0.5 m lithium hexafluorophosphate (LiPF_6_) based electrolyte (solvent, PC/DMC/EC/DEC 1:2:1:2 by volume) sustained 156 cycles and 163 cycles of 100% overcharge per cycle at C/10, respectively, which demonstrates that MPT was relatively stable shuttle molecule offering the overcharge and over‐discharge protection. In the LiFePO_4_/graphite cell, MPT in LiPF_6_ electrolyte showed a better cycling protection performance, 56 cycles of 100% overcharge per cycle compared to that 13 cycles in LiBOB based electrolyte.

#### 2.2.4 Bifunctional Redox Shuttle Additives

**Figure 9:**
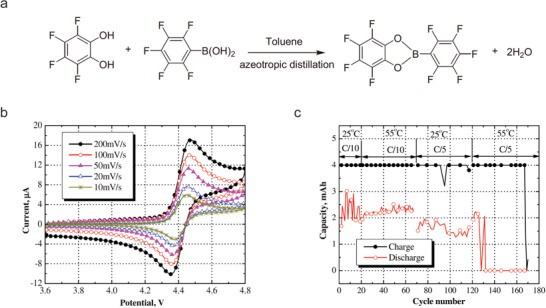
a) Synthesis of 2‐(pentafluorophenyl)‐tetrafluoro‐1,3,2‐benzodioxaborole (PFPTFBB). b) Cyclic voltammogramms of a Pt/Li/Li three‐electrode cell with PFPTFBB in 1.2 m LiPF_6_ (3:7 EC/EMC). c) Charge and discharge capacities of a LiNi_0.8_Co_0.15_Al_0.05_O_2_/graphite cell with 5 wt% PFPTFBB during the whole overcharge test. a) Reproduced with permission.^[^ ^56^ ^]^ Copyright 2010, Elsevier B.V. b,c) Reproduced with permission.^[^ ^50q^ ^]^ Copyright 2006, Elsevier B.V.

Most of the above redox shuttle additives are the electrochemically reversible systems with low redox potentials of less than 4 V, except for DDB series. The redox potential should be increased to over 4.2 V for protecting the LIBs with high voltage cathodes such as LiCoO_2_, LiMn_2_O_4_, and LiNi_0.8_Co_0.15_Al_0.05_O_2_.^[^ ^7^, ^50^ ^]^ The first stable redox shuttle that can be used in the high voltage 4 V class LIBs, 2‐(pentafluorophenyl)‐tetrafluoro‐1,3,2‐benzodioxaborole (PFPTFBB, **Figure** **9**a) was synthesized from the condensation reaction of tetrafluorocatechol (TFC) and pentafluorobenzene boronic acid, which displayed a high redox potential of 4.43 V versus Li/Li^+^ (Figure 9b).^[^ ^50^, ^56^ ^]^ It sustained over 160 cycles with 100% overcharge per cycle under charging rates of C/10 and C/5 at an aggressive condition of 55 °C in a LiNi_0.8_Co_0.15_Al_0.05_O_2_/graphite full cell (Figure 9c). The presence of different functional groups enabled PFPTFB as a bifunctional additive, a redox shuttle for overcharge protection and an anion receptor for extending the life of LIBs. The phenyl group was directly attached to the oxygen atoms making this additive electrochemically reactive; meanwhile the fluorine substitution avoided the polymerization reaction upon oxidation thus increasing the redox potential.^[^ ^52a^ ^]^ The pentafluorophenyl borole functional group enabled PFPTFBB to act as anion receptor to dissolve inorganic components such as LiF from the passivation film, and improve the capacity retention of LIBs. However, PFPTFBB at a high concentration accelerated the LiPF_6_ decomposition. Therefore, a combination of high concentration of PFPTFBB‐F^−^ (converted from PFPTFBB by reacting with LiF) as redox shuttles and low concentration of PFPTFBB as active anion receptors were developed as redox shuttle additives, which demonstrated the same overcharge protection capacity as PFPTFBB but avoided the accelerated decomposition of LiPF_6_ induced from high content of PFPTFBB.^[^ ^29^, ^50^ ^]^

**Figure 10:**
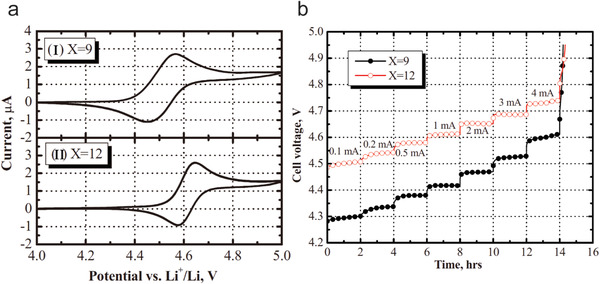
a) Cyclic voltammograms of 0.01 m Li_2_B_12_H_3_F_9_ (I) and 0.01 m Li_2_B_12_F_12_ (II) in 1.0 m LiPF_6_ in a Pt/Li/Li three‐electrode system. b) Voltage profiles as a function of time for mesocarbon microbeads/LiMn_1/3_Ni_1/3_Co_1/3_O_2_ cells in 0.4 m Li_2_B_12_H_3_F_9_ and 0.4 m Li_2_B_12_F_12_ under overcharge at different currents. A solvent of 3:7 EC/EMC was used for all electrolytes. Reproduced with permission.^[^ ^50r^ ^]^ Copyright 2010, ECS – The Electrochemical Society.

A unique family member of high potential redox shuttle additives, lithium borate cluster salts, should be mentioned. They possess the properties of lithium salt and redox shuttle, and thus can act as a lithium ion source as well as provide overcharge protection.^[^ ^50r^ ^]^ The redox potentials of lithium borated cluster salts, Li_2_B_12_H_12‐_ *_x_*F*_x_* (*x*, 1–12), are within a range from 4.2 to 4.7 V (versus Li/Li^+^), which depends on the degree of fluorination. For example, B_12_F_12_^2−^ and B_12_H_3_F_9_^2−^ displayed a redox potential of 4.6 and 4.5 V versus Li/Li^+^, respectively (**Figure** **10**a). The mesocarbon microbeads (MCMB)/LiMn_1/3_Ni_1/3_Co_1/3_O_2_ cells with 1.0 m LiPF_6_ (3:7 EC/DMC) electrolyte containing 0.08 m Li_2_B_12_H_3_F_9_ and Li_2_B_12_F_12_ redox shuttle additives could be charged for up to 40 h to reach full capacity and another 20 h for overcharging at the same current of 0.1 mA. These cells had 20 h long voltage plateaus at 4.4 and 4.55 V under the overcharge condition, respectively. These voltage plateaus originated from the shuttle mechanism of borate clusters that carry most of overcharge current. The shuttle plateau voltage for the MCMB/LiMn_1/3_Ni_1/3_Co_1/3_O_2_ cells in 0.4 m Li_2_B_12_H_3_F_9_ and 0.4 m Li_2_B_12_F_12_ electrolytes increased with the increased current (Figure 10b), and provided a maximum shuttle current of around 4 mA.

To provide an overview of redox shuttle additive‐based electrolytes, structures of redox shuttle additives and their electrochemical properties are summarized in **Table** **2**.

**Table 2:** Overview of redox shuttle additive‐based electrolytes for lithium‐ion batteries

Redox shuttle additives			Redox potential [V^a^ ^)^]	Redox shuttle additive based electrolytes	Overcharge protection	Ref.
Type	Chemicals	Structures				
Inorganic	Iodide/triiodide (I^−^/I_3_^−^)	N/A	≈3.25	0.0119 m lithium iodide in 1.5 m LiAsF_6_/THF^b^ ^)^ electrolyte	N/A	^[^ ^50a,b^ ^]^
Inorganic	Bromide/tribromide (Br^−^/Br_3_^−^)	N/A	≈3.78	19.46 mm solution of lithium bromide in 1.5 m LiAsF_6_/THF electrolyte	N/A	^[^ ^50c^ ^]^
Organic and organometallic	*N*‐DMAMF (dimethylamino‐methylferrocene) (C_13_H_17_FeN)	N/A	3.435	0.3 m DMAMF in 1 m LiAsF_6_ (solvent, 1:1 PC^c^ ^)^/EC)	The Li/Li*_x_*MnO_2_ cells survived up to 100 h during the overcharge.	^[^ ^50e^ ^]^
	2,5‐Di‐*tert*‐butyl‐1,4‐dimethoxybenzene (DDB)	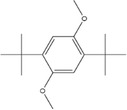	3.9	0.2 m DDB in 0.5 m LiBOB^d^ ^)^ (solvent, 1:2 PC/DEC^e^ ^)^)	The LiFePO_4_/graphite and LiFePO_4_/Li_4/3_Ti_5/3_O_4_ cells survived 200 cycles of 100% overcharge per cycle at C/2 and 1C rate.	^[^ ^50f–h^ ^]^
	3,5‐Di‐*tert*‐butyl‐1,2‐dimethoxybenzene (DBDB)	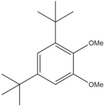	4.2	5 wt% DBDB in 1.2 m LiPF_6_ (solvent, 3:7 EC/EMC)	The LiFePO_4_/Li and LiFePO_4_/graphite cells survived 11 and 20 cycles of 100% overcharge per cycle at C/10 rate, respectively.	^[^ ^50i^ ^]^
	5,7‐Di‐*tert*‐butyl‐1,4‐benzodioxin (DBBD2)	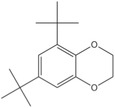	4.17	0.2 m DBBD2 in 1.2 m LiPF_6_ (solvent, 3:7 EC/EMC)	The LiFePO_4_/MCMB^f^ ^)^ full cell survived 24 cycles of 100% overcharge per cycle at C/5 rate.	^[^^]^
	4‐*Tert*‐butyl‐1,2‐dimethoxybenzene (TDB)	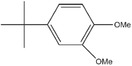	4.1	0.1 m TDB in 1 m LiPF_6_ (solvent, 1:1 EC/DMC)	The LiFePO_4_/Li cell survived 50 cycles of 100% overcharge per cycle at 0.5C rate.	^[^ ^50k^ ^]^
	1,4‐Di‐*tert*‐butyl‐2,5‐bis(2,2,2‐trifluoroethoxybenzene) (DBTFB)	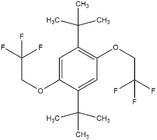	4.25	0.1 m DBTFB in 0.5 m LiPF_6_ (solvent, 1:2:1:2 PC/DMC/EC/DEC)	The LiFePO_4_/Li_4_Ti_5_O_12_ cell survived 170 cycles of 100% overcharge per cycle at C/10 rate.	^[^ ^50l^ ^]^
	Tetraethyl‐2,5‐di‐*tert*‐butyl‐1,4‐phenylene diphosphate (TEDBPDP)	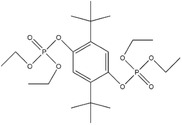	4.8	5 wt% TEDBPDP in 1.2 m LiPF_6_ (solvent, 3:7 EC/EMC)	The LiMn_2_O_4_/Li cell survived 10 cycles of 100% overcharge per cycle at C/10 rate.	^[^ ^50m^ ^]^
	1,4‐Bis[bis(1‐methylethyl)phosphinyl]‐2,5‐dimethoxy‐benzene (BPDB)	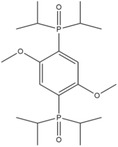	4.5	5 wt% BPDB in 1.2 m LiPF_6_ (solvent, 3:7 EC/EMC)	The LiMn_2_O_4_/MCMB full cell survived 25 cycles of 100% overcharge per cycle at C/10 rate.	^[^ ^50n^ ^]^
	2,2,6,6‐Tetramethylpiperinyloxide (TEMPO)	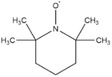	3.5	0.1 m TEMPO in 0.5 m LiBOB (solvent, 1:2:1:2 PC/DMC/EC/DEC)	The Li_4/3_Ti_5/3_O_4_/LiFePO_4_ cell survived 124 cycles of 100% overcharge per cycle at C/10 rate.	^[^ ^50o^ ^]^
	4‐Methoxy‐TEMPO	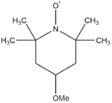	≈3.5–≈3.7	0.1 m 4‐methoxy‐TEMPO in 0.5 m LiBOB (solvent, 1:2:1:2 PC/DMC/EC/DEC)	The Li_4/3_Ti_5/3_O_4_/LiFePO_4_ cell survived 133 cycles of 100% overcharge per cycle at C/10 rate.	^[^ ^50o^ ^]^
	4‐Cyano‐TEMPO	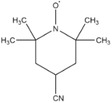	≈3.5– ≈3.7	0.1 m 4‐cyano‐TEMPO in 0.5 m LiBOB (solvent, 1:2:1:2 PC/DMC/EC/DEC)	The Li_4/3_Ti_5/3_O_4_/LiFePO_4_ cell survived 158 cycles of 100% overcharge per cycle at C/10 rate.	^[^ ^50o^ ^]^
	10‐Methylphenothiazine (MPT)	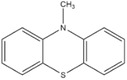	3.55	0.1 m MPT in 0.5 m LiPF_6_ (solvent, 1:2:1:2 PC/DMC/EC/DEC)	The Li_4/3_Ti_5/3_O_4_/LiFePO_4_ cell survived 163 cycles of 100% overcharge per cycle at C/10 rate.	^[^ ^50t^ ^]^
	10‐Ethylphenothiazine (EPT)	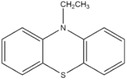	3.65	0.1 m EPT in 0.7 m LiBOB (solvent, 1:2:1:2 PC/DMC/EC/DEC)	The Li_4/3_Ti_5/3_O_4_/LiFePO_4_ cell survived 150 cycles of 100% overcharge per cycle at C/10 rate.	^[^ ^50p^ ^]^
	3‐Chloro‐10‐methylphenothiazine (3‐chloro‐EPT)	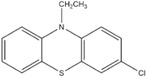	3.75	0.1 m 3‐chloro‐EPT in 0.7 m LiBOB (solvent, 1:2:1:2 PC/DMC/EC/DEC)	The Li_4/3_Ti_5/3_O_4_/LiFePO_4_ cell survived 145 cycles of 100% overcharge per cycle at C/10 rate.	^[^ ^50p^ ^]^
	10‐Isopropylphenothiazine (IPT)	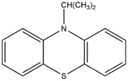	3.80	0.1 m IPT in 0.7 m LiBOB (solvent, 1:2:1:2 PC/DMC/EC/DEC)	The Li_4/3_Ti_5/3_O_4_/LiFePO_4_ cell survived more than 162 cycles of 100% overcharge per cycle at C/10 rate.	^[^ ^50p^ ^]^
Bifunctional	2‐(Pentafluorophenyl)‐tetrafluoro‐1,3,2‐benzodioxaborole (PFPTFBB)	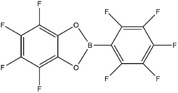	4.43	5 wt% PFPTFBB in 1.2 m LiPF_6_ (solvent, 3:7 EC/EMC)	The LiNi_0.8_Co_0.15_Al_0.05_O_2_/graphite full cell survived 170 cycles of 100% overcharge per cycle at C/5 rate.	^[^ ^50q^ ^]^
	Lithium borate cluster salts (Li_2_B_12_H_12‐_ *_x_*F*_x_* [*x* = 9 or *x* = 12])	N/A	4.5–4.6	0.08 m Li_2_B_12_H_3_F_9_ /Li_2_B_12_F_12_ in 1.0 m LiPF_6_ (solvent, 3:7 EC/EMC)	The MCMB/LiMn_1/3_Ni_1/3_Co_1/3_O_2_ cells survived an extra 20 h for overcharging at a constant current of 0.1 mA.	^[^ ^50r^ ^]^

^a)^V versus Li^+^/Li

^b)^THF, tetrahydrofuran

^c)^PC, propylene carbonate

^d)^LiBOB, lithium bis(oxalato)borate

^e)^DEC, diethyl carbonate

^f)^MCMB, mesocarbon microbeads.

### 2.3 Electrolytes with Flame‐Retardant Additives

#### 2.3.1 Flame Retardant Effects

**Figure 11:**
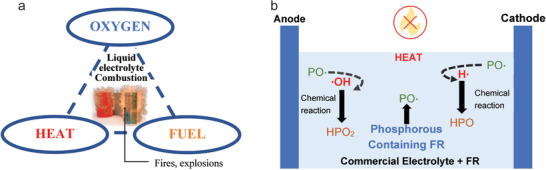
a) Schematic principle of the combustion and explosion of a LIB due to the flammable liquid electrolyte. b) Schematic illustration of the flame‐retardant effect during the thermal runway of LIBs. Reproduced with permission.^[^ ^12d^ ^]^ Copyright 2018, American Association for the Advancement of Science.

Three key elements, heat, fuel and oxygen, are needed to ignite a fire resulting in electrolyte combustion (**Figure** **11**a). Fire can be prevented or extinguished if one of these elements is removed. Flame retardants can inhibit or even prevent combustion by reacting chemically in the condensed phase and/or in the gas phase to eliminate one or more of the key elements^[^ ^57^ ^]^ The concept of using flame retardant additives to increase fire resistance has been applied in many areas from woods, polymers, apparels, modern transportation, to insulation and building materials.^[^ ^58^ ^]^ Both physical and chemical retardants are available.^[^ ^59^ ^]^ They can block the fire process physically via formation of a protective and isolating layer between the condensed and gas phases and prevent the material from igniting. They can also interrupt the chemical reactions in the gas phase by polymer, char, and intumescent formed from FR, which scavenges the radical chain.^[^ ^28^ ^]^ In general, flame retardants suppress burning by terminating the chain reactions involved in combustion.^[^ ^59^, ^60^ ^]^

With LIBs, liquid electrolytes with high flammability act as a fuel for the fire. The addition of flame‐retardant additives into liquid electrolytes is considered to be one of the most effective techniques in providing thermal runaway protection. Therefore, tremendous efforts have been made to develop flame‐retardant (FR) additives to lower the flammability of liquid electrolytes.^[^ ^7^, ^12^, ^28^, ^29^ ^]^ The approach is based on a chemical mechanism as shown in Figure 11b, which involves a chemical radical‐scavenging process. Namely, flame retardants scavenge the highly reactive radicals and terminate the combustion reaction in the gas phase to stop further development.^[^ ^28^, ^29^, ^57^, ^61^ ^]^ Taking the phosphorus‐containing flame retardant as an example, the highly reactive radicals •OH and H• generated from the combustion of the material undergo a chemical reaction with the free phosphorous containing radical PO•, a degradation product present in the gas phase to suppress the exothermic process, and create less reactive radicals for kinetic reduction of the combustion as shown in Equations (4)–(6).^[^ ^12^, ^62^ ^]^ In general, the electrolyte with FR additives not only prevents/slows the propagation of flame/ignition but also decreases the self‐heating rate.^[^ ^28^ ^]^

(4) $$\mathrm{Phosphorus}-\mathrm{based}\;\mathrm{FR}\overset{\mathrm{heat}}{\to }\mathrm{PO}•$$

(5) PO•+H•→HPO

(6) $$\mathrm{PO}•+•\mathrm{OH}\;\to \mathrm{HP}O_{2}$$

The effectiveness of FR additives can be experimentally evaluated using an accelerating rate calorimeter (ARC), self‐extinguishing time (SET) (in other words of the nonflammability of electrolytes),^[^ ^63^ ^]^ and differential scanning calorimetry (DSC). SET is defined as the time of the flame spread resistance.^[^ ^64^ ^]^ Ideal FR additives need to satisfy the following requirements:^[^ ^12^, ^29^ ^]^

1) Physical properties including high solubility, appropriate ionic conductivity, low viscosity, and high boiling point.
2) Chemical stability that does not induce chemical reactions with any components of LIBs.
3) Electrochemical stability—no adverse electrochemical reactions under the operating voltage of LIBs.

Flame‐retardant additives developed to date mainly include organic phosphorous based compounds and organic halogenated compounds.^[^ ^12^, ^61^ ^]^ The halogenated compounds rely on the release of halogen radicals that can participate in the combustion process and react with free‐radical species such as •OH and H• to cascade the combustion of liquid electrolytes.^[^ ^61^ ^]^ However, halogen‐based FR additives are not preferred as they are not environmentally friendly and are harmful to human health.^[^ ^12^, ^65^ ^]^ Phosphorus compounds (i.e., phosphate and phosphite) can efficiently hinder and terminate the chain reactions of •OH or H• radicals during the combustion stage in liquid electrolytes as above discussed. Phosphorous based compounds family as FR additives mainly contain: 1) phosphorus additives, such as trimethyl phosphate (TMP),^[^ ^66^ ^]^ triphenyl phosphate (TPP),^[^ ^67^ ^]^ and ethylene ethyl phosphate (EEP);^[^ ^68^ ^]^ 2) partially fluorinated phosphate FR additives, such as tris(2,2,2‐trifluoroethyl) phosphate(TFP),^[^ ^69^ ^]^ tris(2,2,2‐trifluoroethyl) phosphite (TTFPi),^[^ ^69^, ^70^ ^]^ and bis(2,2,2 trifluoroethyl) methylphosphonate (TFMP);^[^ ^69^, ^71^ ^]^ 3) phosphorous composite additives, such as (ethoxy) pentafluorocyclo‐triphosphazene (PFPN)^[^ ^69^, ^72^ ^]^ and (phenoxy)pentafluorocyclotri‐phosphazene (FPPN).^[^ ^69^, ^73^ ^]^ These FR additives have been systematically investigated over the last two decades. The FR additives in an organic liquid electrolyte can effectively reduce the flammability of the electrolyte due to their ability to scavenge reactive radicals and terminate the combustion reactions under the thermal abuse, thus keeping the LIBs safe.

#### 2.3.2 Phosphorous FR Additives

**Figure 12:**
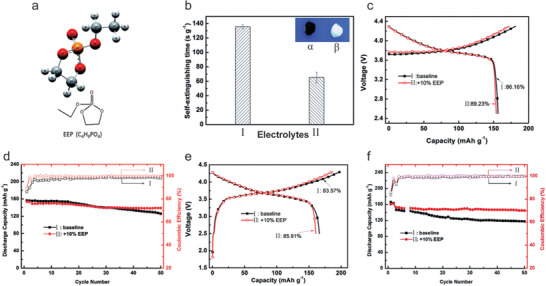
Ethylene ethyl phosphate (EEP) as a FR additive in the electrolytes of 1 m LiPF_6_ in 1:1 EC/DEC containing 0% (I) and 10% EEP (II). a) Chemical structure of EEP molecule. b) SET results of flammability testing for electrolytes (I) and (II); Inset images were scorched cotton ball (*α*) and intact cotton ball (*β*) after the flammability testing. c) The first charge–discharge voltage profiles of LiNi_1/3_Co_1/3_Mn_1/3_O_2_/Li half‐cells. d) Cycling performance of LiNi_1/3_Co_1/3_Mn_1/3_O_2_/Li half‐cells. e) The first charge–discharge voltage profiles of LiNi_1/3_Co_1/3_Mn_1/3_O_2_/graphite full‐cells. f) Cycling performance of LiNi_1/3_Co_1/3_Mn_1/3_O_2_/graphite full cells. Reproduced with permission.^[^ ^68^ ^]^ Copyright 2015, The Royal Society of Chemistry.

Phosphorous compounds are the most popular candidates for flame‐retardant additives in LIBs owing to the advantages of low toxicity, high flame retarding ability, and being environmental friendly.^[^ ^61^ ^]^ These compounds can decompose at high temperature and produce radicals PO• and PO_2_• to capture the highly reactive radicals H• and •OH thus hindering the combustion, and the generation of meta phosphorous acid (HPO_2_) can promote the carbonation reaction as well.^[^ ^22^, ^74^ ^]^ As a result, the combustion of electrolytes in LIBs is terminated. Trimethyl phosphate (TMP) was investigated as FR additive in 2001 by Wang et al.,^[^ ^66^ ^]^ which was the first demonstration of this concept. Electrolytes of 1.0 m LiPF_6_ in EC:PC (1:1) containing 10% TMP and 1.0 m LiPF_6_ in EC:DEC (1:1) containing 25% TMP all showed good cycling performance due to the formation of SEI film on graphite anodes. It may be related to the use of EC in the electrolyte, as EC is a common component to help form a passivating SEI film on graphite anode.^[^ ^75^ ^]^ In addition, TMP increased the thermal stability of electrolytes related with higher boiling point and fewer hydrogen atoms of mixed electrolytes. However, there exists a trade‐off for the electrolyte of 1.0 m LiPF_6_ in pure TMP solvent between the good oxidation stability on the cathode (LiCoO_2_) and poor reduction stability on the graphite anode. The reduction decomposition of TMP on graphite anode occurs at 1.2 V in LIBs. The flame‐retarding triphenyl phosphate (TPP) significantly reduced the flammability of the electrolyte without alternating the flash point as reported by Hyung el al.^[^ ^67^ ^]^ This FR additive in 1 m LiPF_6_ (solvent, 1:1 EC: DEC) was stable up to 5.0 V, which can be effectively and safely used over an operating voltage range of 2.5–4.3 V with minimal negative effect on the cycling performance. The adding of 5 wt% of TPP in the electrolyte significantly improved the safety performance of LIBs, as reflected by the greatly increased onset reaction temperature from 160 to 210 °C, and the exothermic energy generation from the reaction between the fully charged anode and electrolyte was dramatically decreased. Gao et al.^[^ ^68^ ^]^ demonstrated that ethylene ethyl phosphate (EEP) (**Figure** **12**a) not only provided the flame resistance but also the overcharge protection. After adding 10 wt% EEP into the electrolyte of 1 m LiPF_6_ in EC/DEC (1:1), the value of SET (65 s g^−1^) was dropped by 50% compared with the SET for common electrolyte (140 s g^−1^), and there was a ≈10 h buffering time for the voltage reaching 6 V during the overcharging (Figure 12b). In addition, the electrolyte with EEP additives in half cells and full cells all displayed higher initial coulombic efficiency and stable cyclic performance due to the efficient formation of SEI films on both anode and cathode (Figure 12c–f).

#### 2.3.3 Partially Fluorinated Phosphate FR Additives

Partially fluorinated phosphate FR additives offered better cycling performance and FR effectiveness compared to phosphorous additives as reported by Murmann et al.^[^ ^76^ ^]^ These additives effectively reduced the boiling temperature and viscosity of electrolyte as well as restrained the flammability. Reactive radicals H• were reduced or captured through fluorine atoms forming a non‐flammable electrolyte. Tris(2,2,2‐trifluoroethyl) phosphate (TFP), also named as fluorinated alkylphosphate, was developed as a FR additive for LIB electrolytes by Ding et al.^[^ ^69a^ ^]^ and Xu et al.^[^ ^69b–d^ ^]^ The adding of this additive (TFP) into the electrolyte of LiPF_6_ in EC/PC/EMC (3:3:4) displayed good flame retarding effect: a continuous decreasing in the dielectric constant, a continuous increase of *T*_g_ (glass transition temperature) and *θ*_b_ (boiling point).^[^ ^69a^ ^]^ In addition, Xu et al.^[^ ^69b–d^ ^]^ showed that a commercial electrolyte (1.0 m LiPF_6_ in EC/EMC) containing less than 20% of TFP not only increased the flame retarding ability but also improved the electrochemical performance including an increased capacity retention and utilization.

**Figure 13:**
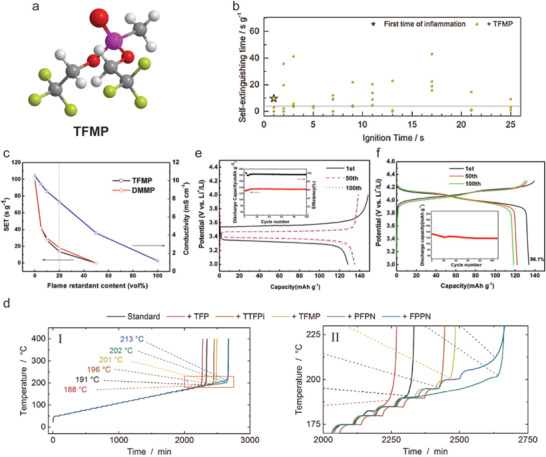
Bis(2,2,2‐trifluoroethyl) methylphosphonate (TFMP) as a FR additive in an electrolyte of 1 m LiPF_6_ in EC/DMC. a) Chemical structure of TFMP molecule. b) Self‐extinguishing time as a function of the ignition time of the electrolyte with 5 wt% TFMP. The ignition time is the time in which the sample was exposed directly to a flame. c) The SET results and conductivity of electrolytes containing different concentration of TFMP and DMMP. d) Temperature results in dependency of time (I) and the expanded view (II) for the electrolyte with or without FR additives (standard electrolyte, black; TFP, pink; TTFPi, red; TFMP, yellow; PFPN, green; FPPN, blue) during the heat‐wait‐search experiments. (e,f), The electrochemical performance of LiFePO_4_/Li half‐cells (e) and LiMn_2_O_4_/Li half‐cells (f) in the electrolyte with 20 vol% of TFMP. b,d) Reproduced with permission.^[^ ^69f^ ^]^ Copyright 2018, Wiley‐VCH. c,e,f) Reproduced with permission.^[^ ^71^ ^]^ Copyright 2014, Elsevier Ltd.

A novel fluorinated alkyl phosphonate FR additive, bis(2,2,2‐trifluoroethyl) methylphosphonate (TFMP, **Figure** **13**a) was developed as a bifunctional FR additive with good FR efficiency and minimal impact on the electrochemical performance by Zeng et al.^[^ ^71^ ^]^ The addition of 20% TFMP into the electrolyte of 1 m LiPF_6_ in EC/DMC suppressed the flammability (Figure 13b), and the SET was sharply decreased from around 100 to 13.6 s g^−1^, a substantial reduction of 86% (Figure 13c). In addition, the onset temperature of the thermal runaway was shifted to 201 from 191 °C after the addition of 5 wt% TFMP (Figure 13d). This low concentration of TFMP had minor impacts on the electrochemical performance of graphite anode, which demonstrated a higher initial charge and discharge capacity than in the original electrolyte, but with a lower initial coulombic efficiency of 68.3% compared to that 77% due to the decomposition of TFMP, an excellent capacity retention of 93% over 100 cycle. The LiFePO_4_ cathode showed excellent electrochemical performance in the electrolyte with 20 vol% TFMP, as the reversible capacity incrementally increased from 128 to 139 mAh g^−1^ over 100 cycles (Figure 13e). Using the LiMn_2_O_4_ electrode good cycling stability, an initial reversible capacity of 134 mAh g^−1^ and a capacity retention of 89% over 100 cycles were obtained (Figure 13f). These results evidenced good compatibility between the electrolyte of LiPF_6_ in EC/DMC containing TFMP FR and electrodes, including LiFePO_4_ and LiMn_2_O_4_ cathodes as well as graphite anode.

#### 2.3.4 Phosphorous Composite FR Additives

**Figure 14:**
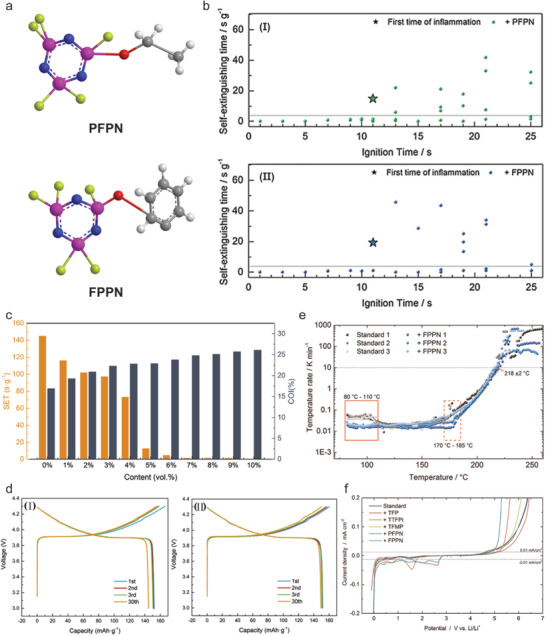
a) Chemical structure of flame‐retardant additives of ethoxy (pentafluoro) cyclotriphosphazene (PFPN) and phenoxy (pentafluoro) cyclotriphosphazene (FPPN). b) Self‐extinguishing time as a function of the ignition time of the standard electrolyte containing 5 wt% PFPN (I) and 5 wt% FPPN (II). The ignition time is the time when the sample was exposed directly to a flame. c) The results of SET and COI for PFPN in 1.0 m LiPF_6_ (1:1 EC/DMC) electrolyte. d) The cycling performance of LiCoO_2_/Li half cells in 1.0 m LiPF_6_ (1:1 EC/DMC) electrolyte containing I) 0% PFPN and II) 5 vol% PFPN. e) Self‐heating rate as a function of temperature for heat‐wait‐search measurement of a 5 Ah cell in the commercial electrolyte without or with 5 wt% FPPN. The red rectangle indicates the temperature range where FPPN decreases the self‐heating rate. The red dash rectangle marks the area of temperature range where the temperature rate of all cell samples increases. The black horizontal dash line indicates the thermal runaway threshold value of 10 K·min^−1^. f) Electrochemical stability window for the commercial electrolyte (1.0 m LiPF_6_ in 1:1 EC/DMC electrolyte) with 0 wt% FR [black], and 5 wt% FR including TFP [pink], TTFPi [red], TFMP [yellow], PFPN [green], and FPPN [blue]. b) Reproduced with permission.^[^ ^69f^ ^]^ Copyright 2018, Wiley‐VCH. c,d,f) Reproduced with permission.^[^ ^72^ ^]^ Copyright 2018, Elsevier B.V. e) Reproduced with permission.^[^ ^73^ ^]^ Copyright 2018, Wiley‐VCH.

Phosphorous composites used as FR additives are formed by combining two or more different types of phosphorous‐containing flame retardants. The addition of composite additives into the electrolyte can achieve superior properties compared to single flame retardant such as better solubility and electrolyte compatibility with electrodes. From a comparative investigation of five flame retardant additives (TFP, TTFPi, TFMP, PFPN and FPPN) in 1 m LiPF_6_ in EC:DMC (1:1),^[^ ^69e,f^ ^]^ it was found that the fluorine content of additives played a minor role in the flame retardant effectiveness, and it synergized with the phosphorus and nitrogen contents to enhance fire protection. Li et al.^[^ ^72^ ^]^ synthesized ethoxy (pentafluoro) cyclotriphosphazene (PFPN, **Figure** **14**a) as a composite FR additive. A new non‐flammable multifunctional electrolyte was formed after the adding of 5 vol% of PFPN into the commercial electrolyte, which could improve the low temperature performance of LiCoO_2_ cathode as well as decrease the electrode polarization due to the reduced charge transfer resistance. Besides, phenoxy (pentafluoro) cyclotriphosphazene (FPPN, Figure 14a) showed better FR performance and excellent electrochemical properties as well. Dagger et al.^[^ ^73^ ^]^ studied the thermal abuse resistant properties of a 5 Ah battery with a standard electrolyte containing fire retardant additives. The electrolyte containing 5 wt% phosphazene composited FR (I: PFPN, II: FPPN) could completely suppress the flammability for around 11s (Figure 14b). However, after the ignition for 10s, the concentration of FR additives was reduced to the level when FR additives no longer suppressed the flammability and the self‐extinguishing times of PFPN and FPPN were not the same (Figure 14b). The PF series electrolytes, electrolytes with different PFPN contents, displayed a sharp decrease in SET with the increasing concentration of PFPN (Figure 14c) evidencing the high flame retardant efficiency. The electrolyte containing 5 vol% PFPN demonstrated a SET of 12.38 s g^−1^ and a COI of 22.9%, evidencing its non‐flammable property. The LiCoO_2_/Li half‐cells with the standard electrolyte and 5 vol% PFPN added electrolyte displayed similar charge and discharge curves (Figure 14d), and a capacity retention rate of 95.2% and 99.14% after discharging for 30 cycles at 0.1 C, respectively. It is clear that the presence of PFPN in the standard electrolyte improved the capacity retention, as the formation of more electrophilic SEI accelerated the transportation of lithium ion and inhibited the irreversible decomposition. The SET of cells with electrolyte containing 5 wt% FPPN was significantly reduced over the temperature between 80 and 110 °C compared to the cells with standard electrolyte (Figure 14e), indicating a lower self‐heating rate to delay the thermal runaway. The addition of FPPN and TTFPi into the standard electrolyte negatively influenced the oxidative stability of electrolyte, but the PFPN containing electrolyte retained the similar stability as standard electrolyte (Figure 14f). During the CV test, all additives showed partial electrolyte decomposition towards higher potentials and the decomposition reactions only happened in the first cycle. The LiNi_1/3_Mn_1/3_Co_1/3_O_2_ (NMC111) based working electrode in the FPPN added electrolyte displayed a huge current peak shift to higher protentional in the first cycle of the cyclic voltammetry scans. However, there is no further electrolyte decomposition in following cycles.^[^ ^72^ ^]^ Besides, PFPN and FPPN provided the best cycle performances with low self‐discharge behavior and increased thermal stability of the delithiated cathode material.^[^ ^69e^ ^]^

**Table** **3** presents an overview of flame‐retardant additive‐based electrolytes, which include the additives used, the flame retardant effect, and their electrochemical performance in LIBs.

**Table 3:** Overview of flame‐retardant additive‐based electrolytes for lithium‐ion batteries

Flame‐retardant additives		Flame retardant additive‐based electrolytes	Cell properties		Ref.
Type	Chemicals		Flame retardant effect	Electrochemical performance	
Phosphorus additives	Trimethyl phosphate [(CH_3_)_3_PO_4_, TMP]	10% TMP in 1 m LiPF_6_ (solvent, 1:1 EC/PC)	Improved the thermal stability of LiCoO_2_/graphite cell, reflected as the lowered heat flow at high temperature.	Good oxidation stability on LiCoO_2_ cathode; the Li/graphite cell showed good cycling performance.	^[^ ^66^ ^]^
	Triphenyl phosphate [(C_6_H_5_O)_3_PO, TPP]	5% TPP in 1 m LiPF_6_ (solvent, 1:1 EC/DEC)	Excellent thermal stability; self‐heating rate of less than 0.1 °C min^−1^; high onset temperature of 210 °C.	Electrochemical stable window up to 5.0 V; the LiNi_0.8_Co_0.2_O_2_/graphite cell showed a slightly decreased capacity after 150 cycles.	^[^ ^67^ ^]^
	Ethylene ethyl phosphate [C_4_H_9_PO_4_, EEP]	10% EEP in 1 m LiPF_6_ (solvent, 1:1 EC/DEC)	Flammability of electrolyte was inhibited as the SET^a^ ^)^ was reduced from 140 to 65 s g^−1^.	The LiNi_1/3_Co_1/3_Mn_1/3_O_2_/Li half cell and the full cell with graphite anode displayed high initial coulombic efficiency and stable cyclic performance.	^[^ ^68^ ^]^
Partially fluorinated phosphate additives	Tris(2,2,2‐trifluoroethyl) phosphate [C_6_H_6_F_9_O_4_P, TFP]	20% TFP in 1 m LiPF_6_ (solvent, 1:1 EC/EMC)	A non‐flammable electrolyte with a SET of around 6 s g^−1^	The nickel oxide‐based/graphite cell displayed long‐term stability, improved capacity retention and capacity utilization.	^[^ ^69a–d^ ^]^
	Tris(2,2,2‐trifluoroethyl) phosphite [(CF_3_CH_2_O)_3_P, TTFPi]	15% TTFPi in 1 m LiPF_6_ (solvent, 3:3:4 by weight, PC/EC/EMC)	A non‐flammable electrolyte with a SET of around zero	Cycling performance of the lithium nickel‐based mixed oxide/graphite cell was improved; the graphite/Li cell showed increased coulombic efficiency.	^[^ ^69^, ^70^ ^]^
	Bis(2,2,2‐trifluoroethyl) methylphosphonate [C_5_H_7_F_6_O_3_P, TFMP]	20 vol% TFMP in 1 m LiPF_6_ (solvent, 1:1 EC/DMC)	A non‐flammable electrolyte with a SET of 13.60 s g^−1^	The graphite/Li cell showed a capacity of 346 mAh g^−1^ and a capacity retention of 93% after 100 cycles; LiFePO_4_/Li and LiMn_2_O_4_/Li cells showed good cycling stability.	^[^ ^71^ ^]^
Phosphorous composite additives	Ethoxy (pentafluoro) cyclotriphosphazene [C_2_H_5_F_5_N_3_OP_3_, PFPN]	5 vol% PFPN in 1 m LiPF_6_ (solvent, 1:1 EC/DMC)	A non‐flammable electrolyte with a SET of 12.38 s g^−1^; critical oxygen index of 22.	The LiCoO_2_/Li cell showed good cycling stability and capacity retention.	^[^ ^72^ ^]^
	Phenoxy (pentafluoro) cyclotriphosphazene [C_6_H_5_F_5_N_3_OP_3_, FPPN]	5% FPPN in 1 m LiPF_6_ (solvent, 1:1 EC/DMC)	Non‐flammable time of 10s; self‐hearting rate was reduced in the temperature range between 80 and 110 °C.	N/A	^[^ ^73^ ^]^

^a)^SET, self‐extinguishing time.

## 3 Summary and Perspectives

Mechanical, electrochemical, and thermal abuse comprise the safe operation of lithium‐ion batteries. Such abuse can cause thermal runaway incidents resulting in fire or explosions. Safety issues currently limit the development of advanced LIBs, especially for the use as large‐scale energy storage. Different strategies have been applied to prevent/terminate thermal runaway incidents. The use of intrinsically safe materials is considered as the “ultimate” solution, which is the driving force for the development of abuse‐resistant electrolytes. This type of electrolyte can be prepared by adding a small amount of additives/fillers into the commonly used liquid electrolyte. In this review, we have presented and discussed three types of abuse‐tolerant electrolytes:STEs, electrolytes with redox shuttle additives, and electrolytes with flame retardant additives.

### 3.1 Shear Thickening Electrolytes

STEs can be regarded as a special type of STFs and provide the protection against the dynamic impact. They function as conventional liquid electrolyte with high ionic conductivity and good interfacial compatibility with electrodes in a normal state. STEs transform into semisolid or solid phase due to the shear thickening effect under the physical impact, which can dissipate the energy impacts and tolerate the mechanical abuse providing the protection. This is a transient and reversible phase transformation, which returns to the original state after the removal of impact. 0D nanoparticles and 1D nanomaterials are two major types of fillers added in STEs. The fillers shape, for example, AR, influences on the rheological behaviors. With the increased AR, both the maximum packaging density and interparticle attraction are decreased resulting in reduced critical volume fraction for shear thickening performance and a consequently increased energy density.

The development of STEs is still at the early stage. Their practical use in high performance LIBs currently face several key challenges, such as the capability to endure both low energy and high energy impact, the achieving of high ionic transport and high impact resistance, and the compatibility with electrodes especially for high impact resistant electrolytes. There also exists fundamental questions to answer such as key factors affecting the shear thickening effectiveness in the electrolyte with high concentration of fillers and the ionic transport properties during the shear thickening process. A deep understanding of the working mechanism of shear thickening behavior and the correlation with the composition of STEs are required in order to enable the design and fabrication of new STEs and promote the practical implementation. The use of in situ and operando characterization techniques are powerful tools in this regard.^[^ ^77^ ^]^

### 3.2 Electrolytes with Redox Shuttle Additives

Overcharge is one of the most common causes of thermal runaway incidents in LIBs. The addition of redox shuttle additives into the electrolyte can improve the safety of LIBs under overcharge conditions by preventing decomposition reactions. Redox shuttle additives mainly include inorganic redox shuttles, organic redox shuttles (e.g., organometallic metallocenes, phenothiazines, triphenylamines, dimethoxybenzenes, and their derivatives), and bifunctional redox shuttles. The key criteria for selecting a redox shuttle additive for overcharge protection mainly include: 1) they should have slightly higher potential window than the end‐of‐charge potential of the cathode, but not exceed the operating electrochemical window of electrolytes; 2) the redox mechanism is highly reversible; 3) stable radical cations are formed to diffuse across the electrolyte, which prevent the overcharge induced decomposition reactions of all battery components at high voltages via shunting the overcharge current.

It is a challenge to find a practical redox shuttle additive that can possess all of the desirable characteristics such as appropriate redox potential, good solubility, and diffusion coefficient, in order to provide high electrochemical performance, suffer large current overcharge, and maintain the chemical and electrochemical stability for long‐term overcharge protection. Particularly, it is hard to predict the reactivity of the electro‐deficient species and identify kinetically stable redox shuttles in battery electrolyte for stable and high voltage redox shuttles.^[^ ^78^ ^]^ In addition, the development of redox shuttle additives with high redox potentials (4.5–4.8 V, or higher) is in an urgent need to protect high‐voltage cathode materials for the state‐of‐the‐art high‐performance LIBs.

### 3.3 Electrolytes with Fire‐Retardant Additives

Thermal runaway incidents occur more rapidly when LIBs experience thermal abuse. The addition of flame‐retardant additives into the electrolyte can suppress the flammability and improve thermal stability of LIBs. Specifically, flame retardant additives can scavenge highly reactive radicals and terminate the chain propagation reactions involved in combustion. To qualify for being a FR additive, it needs to be of high anti‐flammability, thermal stability, and good compatibility with electrolytes and electrodes. Phosphorus based compounds are the mostly used FR additives, including phosphorus additives, partially fluorinated phosphate additives, and phosphorous composite additives. Among them, phosphorous composite additives have demonstrated the high retardant efficiency and good electrochemical performance.

Currently, the key challenges facing FR additives mainly include: 1) the reductive decomposition and the compatibility of the formed electrolytes with electrodes for phosphorus FR additives; 2) the high retardant efficiency and low cost for partially fluorinated phosphate additives; 3) the low cost and efficient methods to synthesize phosphorous composite FR additives. Most importantly, the development of FR additives needs to consider the balance between the retardant effect and the electrochemical performance.

It should be noted that currently used additives in electrolytes only provide one type of protection. It is ideal to develop new fascinating materials that possess all the abuse‐resistant properties in electrolytes, thus to greatly improve the safety issues of LIBs and promote the use of lithium‐ion batteries in grid energy systems and create a more sustainable society. Bifunctional additives containing flame‐retardant functional groups (e.g., fluorine‐, phosphorus‐, sulfur‐, or boron‐containing functional groups) and polymerizable redox shuttle functionalities could be synthesized as a fascinating additive candidate (e.g., (4‐methoxy)‐phenoxy pentafluorocyclotriphosphazene,^[^ ^79^ ^]^ 1‐diphenylphosphoryloxy‐4‐methylbenzene^[^ ^80^ ^]^), which may enable the formation of electrolytes that hinder the flame propagation/ignition of electrolytes due to the radical absorption and oxygen isolation mechanism as well as limit the battery voltage within a safe range against overcharge by forming a passivation layer.^[^ ^79^, ^80^, ^81^ ^]^ In the past, a time‐consuming and inefficient trial‐and‐error approach has been applied to develop specific additives for use in the abuse‐resistant electrolytes, which is an individual selection method. Currently, high‐throughput screening techniques have become popular and powerful in the discovery of novel materials, the prediction of physical and chemical properties based on big data owing to their prediction accuracy and low cost for computation.^[^ ^29^, ^82^ ^]^ Machine learning can fast and accurately predict varieties of chemistries of additives or fillers with desirable properties for multifunctional electrolytes.^[^ ^83^ ^]^ It may become a reality that additives can overcome all the above‐mentioned challenges without any side reactions. It is also envisioned that the progress can be promoted for developing abuse‐resistant electrolyte for safe LIBs system with the help of in situ and operando characterization and machine‐learning techniques.^[^ ^77^, ^84^ ^]^

## Conflict of Interest

The authors declare no conflict of interest.

## References

[1] a) A. Q. Al‐Shetwi , M. A. Hannan , K. P. Jern , M. Mansur , T. M. I. Mahlia , J. Cleaner Prod. 2020, 253, 119831;

[2] a) S. Abada , G. Marlair , A. Lecocq , M. Petit , V. Sauvant‐Moynot , F. Huet , J. Power Sources 2016, 306, 178;

[3] a) Z. Yao , W. Tang , X. Wang , C. Wang , C. Yang , C. Fan , J. Power Sources 2020, 448, 227456;

[4] a) Rechargeable Lithium Batteries: From Fundamentals to Applications, (Ed: A. A. Franco ), Woodhead Publishing, Sawston, UK 2015;

[5] a) A. Ramanan , Resonance 2019, 24, 1381;

[6] Lithium Ion Battery Market to 2025 ‐ Global Analysis and Forecasts Type, by Power Capacity, by Application, Research and Markets, 2018.

[7] a) P. G. Balakrishnan , R. Ramesh , T. P. Kumar , J. Power Sources 2006, 155, 401;

[8] X. Feng , M. Ouyang , X. Liu , L. Lu , Y. Xia , X. He , Energy Storage Mater. 2018, 10, 246.

[9] TechRadar , Here's why the Samsung Galaxy Note 7 batteries caught fire and exploded, https://www.techradar.com/au/news/samsung‐galaxy‐note‐7‐battery‐fires‐heres‐why‐they‐exploded (accessed: April 2019).

[10] Japan Transport Safety Board , in Aircraft Serious Incident Investigation Report, (Eds: N. Goto , S. Endo , T. Ishikawa , S. Tamura , Y. Shuto , K. Tanaka ), Japan Transport Safety Board, Chiyoda‐ku, Japan 2014.

[11] a) D. H. Doughty , E. P. Roth , Electrochem. Soc. Interface 2012, 21, 37;

[12] a) D. Ouyang , M. Chen , Q. Huang , J. Weng , Z. Wang , J. Wang , Appl. Sci. 2019, 9, 2483;

[13] D. Ren , X. Feng , L. Lu , X. He , M. Ouyang , Appl. Energy 2019, 250, 323.

[14] Z. Wang , H. Yang , Y. Li , G. Wang , J. Wang , J. Hazard. Mater. 2019, 379, 120730. 31252342 10.1016/j.jhazmat.2019.06.007

[15] a) J.‐H. Kim , K.‐H. Lee , D.‐C. Ko , S.‐B. Lee , B.‐M. Kim , J. Mech. Sci. Technol. 2017, 31, 2505;

[16] a) A. Augeard , T. Singo , P. Desprez , F. Perisse , S. Menecier , M. Abbaoui , presented at 2014 IEEE 60th Holm Conf. Electrical Contacts (Holm), New Orleans, LA 2014;

[17] A. Rahali , M. Ouremchi , A. Elboutahiri , K. Elkhadiri , A. Tahiri , H. Qjidaa , in 2019 7th Mediterranean Congress of Telecommunications (CMT), IEEE, New York 2019.

[18] a) D. Chen , J. Jiang , G.‐H. Kim , C. Yang , A. Pesaran , Appl. Therm. Eng. 2016, 94, 846;

[19] a) Y. Wang , W.‐H. Zhong , ChemElectroChem 2015, 2, 22;

[20] D. Aurbach , Y. Talyosef , B. Markovsky , E. Markevich , E. Zinigrad , L. Asraf , J. S. Gnanaraj , H.‐J. Kim , Electrochim. Acta 2004, 50, 247.

[21] a) H. P. Zhang , P. Zhang , Z. H. Li , M. Sun , Y. P. Wu , H. Q. Wu , Electrochem. Commun. 2007, 9, 1700;

[22] a) A. Manuel Stephan , Eur. Polym. J. 2006, 42, 21;

[23] a) Y.‐Z. Sun , J.‐Q. Huang , C.‐Z. Zhao , Q. Zhang , Sci. China: Chem. 2017, 60, 1508;

[24] a) H. Srour , L. Chancelier , E. Bolimowska , T. Gutel , S. Mailley , H. Rouault , C. C. Santini , J. Appl. Electrochem. 2016, 46, 149;

[25] a) X. Zuo , X.‐M. Liu , F. Cai , H. Yang , X.‐D. Shen , G. Liu , J. Power Sources 2013, 239, 111;

[26] a) D. Zhou , X. Mei , J. Ouyang , J. Phys. Chem. C 2011, 115, 16688;

[27] a) M. Kirchhöfer , J. Von Zamory , E. Paillard , S. Passerini , Int. J. Mol. Sci. 2014, 15, 14868; 25153637 10.3390/ijms150814868 PMC4159887

[28] S. S. Zhang , J. Power Sources 2006, 162, 1379.

[29] A. M. Haregewoin , A. S. Wotango , B.‐J. Hwang , Energy Environ. Sci. 2016, 9, 1955.

[30] K. Shu , C. Wang , W. Li , T. Bussell , J. Ding , Curr. Opin. Electrochem. 2020, 21, 297.

[31] H. A. Barnes , J. Rheol. 1989, 33, 329.

[32] a) N. J. Wagner , J. F. Brady , Phys. Today 2009, 62, 27;

[33] a) X. Li , H. L. Cao , S. Gao , F. Y. Pan , L. Q. Weng , S. H. Song , Y. D. Huang , Plast., Rubber Compos. 2008, 37, 223;

[34] a) X. Z. Zhang , W. H. Li , X. L. Gong , Smart Mater. Struct. 2008, 17, 035027;

[35] W. C. I. P. Hunt , H. Craig (Mobil Oil Corporation), *US4982792A*, 1991.

[36] B. J. Maranzano , N. J. Wagner , J. Chem. Phys. 2002, 117, 10291.

[37] a) X. Zhang , W. Li , X. Gong , Smart Mater. Struct. 2008, 17, 1.

[38] a) C. Pfaffenhuber , M. Goebel , J. Popovic , J. Maier , Phys. Chem. Chem. Phys. 2013, 15, 18318; 24080900 10.1039/c3cp53124d

[39] a) G. Bossies , Y. Grasselli , A. Meunier , O. Volkova , J. Intell. Mater. Syst. Struct. 2018, 29, 5;

[40] S. Gürgen , M. C. Kuşhan , W. Li , Prog. Polym. Sci. 2017, 75, 48.

[41] J. Ding , T. Tian , Q. Meng , Z. Guo , W. Li , P. Zhang , F. T. Ciacchi , J. Huang , W. Yang , Sci. Rep. 2013, 3, 1. 10.1038/srep02485 PMC6505397 23962885

[42] a) G. M. Veith , B. L. Armstrong , H. Wang , S. Kalnaus , W. E. Tenhaeff , M. L. Patterson , ACS Energy Lett. 2017, 2, 2084;

[43] Y. Ye , J. L. Lutkenhaus , H. Xiao , K. Reaves , B. McCulloch , J. F. Mike , ACS Appl. Nano Mater. 2018, 1, 2774.

[44] V. T. O'Brien , M. E. Mackay , J. Rheol. 2002, 46, 557.

[45] I.‐N. Yoon , H.‐k. Song , J. Won , Y. S. Kang , J. Phys. Chem. C 2014, 118, 3918.

[46] K. Liu , C.‐F. Cheng , L. Zhou , F. Zou , W. Liang , M. Wang , Y. Zhu , J. Power Sources 2019, 423, 297.

[47] L. Zhang , Z. Zhang , K. Amine , in Lithium Ion Batteries—New Developments, (Ed: I. Belharouak ), IntechOpen, London 2012.

[48] J. Garche , K. Brandt , in Electrochemical Power Sources: Fundamentals, Systems, and Applications, (Eds: J. Garche , K. Brandt ), Elsevier, New York 2019, p. 143.

[49] a) F. Zhang , T. Geng , F. Peng , D. Zhao , N. Zhang , H. Zhang , S. Li , ChemElectroChem 2019, 6, 731;

[50] a) W. K. Behl , D.‐T. Chin , J. Electrochem. Soc. 1988, 135, 16;

[51] a) L. R. Meites , B. Elinore , P. Zuman , in CRC Handbook Series in Organic Electrochemistry, CRC Press, Cleveland 1977;

[52] a) Z. Chen , Q. Wang , K. Amine , J. Electrochem. Soc. 2006, 153, A2215;

[53] a) Z. Wang , J. Yuan , X. Zhu , H. Wang , L. Huang , Y. Wang , S. Xu , J. Energy Chem. 2021, 55, 484;

[54] G. GirishKumar , W. H. Bailey , B. K. Peterson , W. J. Casteel , J. Electrochem. Soc. 2011, 158, A146.

[55] M. Taggougui , B. Carré , P. Willmann , D. Lemordant , J. Power Sources 2007, 174, 643.

[56] W. Weng , Z. Zhang , J. A. Schlueter , P. C. Redfern , L. A. Curtiss , K. Amine , J. Power Sources 2011, 196, 2171.

[57] a) A. Mack , FR Mechanisims, http://fr.polymerinsights.com/home/mechanisims (accessed: May 2020);

[58] a) P. Kiliaris , C. D. Papaspyrides , in Polymer Green Flame Retardants, (Eds: C. D. Papaspyrides , P. Kiliaris ), Elsevier, Amsterdam 2014;

[59] C. M. C. Pereira , M. S. S. Martins , in Polymer Green Flame Retardants, (Eds: C. D. Papaspyrides , P. Kiliaris ), Elsevier, Amsterdam 2014, p. 551.

[60] P. Nousiainen , S. Heidari , Makromol. Chem., Macromol. Symp. 1993, 74, 41.

[61] F. Laoutid , L. Bonnaud , M. Alexandre , J. M. Lopez‐Cuesta , P. Dubois , Mater. Sci. Eng., R 2009, 63, 100.

[62] S. V. Levchik , in Flame Retardant Polymer Nanocomposites, (Eds: A. B. Morgan , C. A. Wilkie ), Wiley, Boboken, NJ 2006, Ch. 1.

[63] T. Dagger , C. Lürenbaum , F. M. Schappacher , M. Winter , J. Power Sources 2017, 342, 266.

[64] F. Carosio , L. Maddalena , J. Gomez , G. Saracco , A. Fina , Adv. Mater. Interfaces 2018, 5, 1801288.

[65] M. Monisha , P. Preetham , A. Ghosh , A. Kumar , S. Zafar , S. Mitra , B. Lochab , Energy Storage Mater. 2020, 29, 350.

[66] X. M. Wang , E. Yasukawa , S. Kasuya , J. Electrochem. Soc. 2001, 148, A1058.

[67] Y. E. Hyung , D. R. Vissers , K. Amine , J. Power Sources 2003, 119, 383.

[68] D. Gao , J. B. Xu , M. Lin , Q. Xu , C. F. Ma , H. F. Xiang , RSC Adv. 2015, 5, 17566.

[69] a) M. S. Ding , K. Xu , T. R. Jow , J. Electrochem. Soc. 2002, 149, A1489;

[70] S. S. Zhang , K. Xu , T. R. Jow , J. Power Sources 2003, 113, 166.

[71] Z. Zeng , X. Jiang , B. Wu , L. Xiao , X. Ai , H. Yang , Y. Cao , Electrochim. Acta 2014, 129, 300.

[72] X. Li , W. Li , L. Chen , Y. Lu , Y. Su , L. Bao , J. Wang , R. Chen , S. Chen , F. Wu , J. Power Sources 2018, 378, 707.

[73] T. Dagger , V. Meier , S. Hildebrand , D. Brüggemann , M. Winter , F. M. Schappacher , Energy Technol. 2018, 6, 2001.

[74] a) H. F. Xiang , H. Y. Xu , Z. Z. Wang , C. H. Chen , J. Power Sources 2007, 173, 562;

[75] a) D. Aurbach , Y. Ein‐Eli , B. Markovsky , A. Zaban , S. Luski , Y. Carmeli , H. Yamin , J. Electrochem. Soc. 1995, 142, 2882;

[76] P. Murmann , X. Mönnighoff , N. von Aspern , P. Janssen , N. Kalinovich , M. Shevchuk , O. Kazakova , G.‐V. Röschenthaler , I. Cekic‐Laskovic , M. Winter , J. Electrochem. Soc. 2016, 163, A751.

[77] a) D. Liu , Z. Shadike , R. Lin , K. Qian , H. Li , K. Li , S. Wang , Q. Yu , M. Liu , S. Ganapathy , X. Qin , Q.‐H. Yang , M. Wagemaker , F. Kang , X.‐Q. Yang , B. Li , Adv. Mater. 2019, 31, 1806620; 10.1002/adma.201806620 31099081

[78] S. Ergun , C. F. Elliott , A. P. Kaur , S. R. Parkin , S. A. Odom , J. Phys. Chem. C 2014, 118, 14824.

[79] T. Huang , X. Z. Zheng , G. F. Fang , Y. Pan , W. G. Wang , M. X. Wu , RSC Adv. 2017, 7, 47775.

[80] P. Yan , Y. Zhu , X. Pan , H. Ji , Int. J. Energy Res. 2021, 45, 2776.

[81] T. van Ree , Curr. Opin. Electrochem. 2020, 21, 22.

[82] a) M. A. Makeev , N. N. Rajput , Curr. Opin. Chem. Eng. 2019, 23, 58;

[83] a) L. D. Ellis , S. Buteau , S. G. Hames , L. M. Thompson , D. S. Hall , J. R. Dahn , J. Electrochem. Soc. 2018, 165, A256;

[84] L. Trahey , F. R. Brushett , N. P. Balsara , G. Ceder , L. Cheng , Y.‐M. Chiang , N. T. Hahn , B. J. Ingram , S. D. Minteer , J. S. Moore , K. T. Mueller , L. F. Nazar , K. A. Persson , D. J. Siegel , K. Xu , K. R. Zavadil , V. Srinivasan , G. W. Crabtree , Proc. Natl. Acad. Sci. USA 2020, 117, 12550. 32513683 10.1073/pnas.1821672117 PMC7293617

